# Development of Resistance in *Escherichia coli* ATCC25922 under Exposure of Sub-Inhibitory Concentration of Olaquindox

**DOI:** 10.3390/antibiotics9110791

**Published:** 2020-11-10

**Authors:** Yufeng Gu, Shuge Wang, Lulu Huang, Wei Sa, Jun Li, Junhong Huang, Menghong Dai, Guyue Cheng

**Affiliations:** 1 College of Veterinary Medicine, Huazhong Agricultural University, Wuhan 430070, China; guyufeng@webmail.hzau.edu.cn (Y.G.); shugewang@webmail.hzau.edu.cn (S.W.); huanglu@webmail.hzau.edu.cn (L.H.); weisa566@gmail.com (W.S.); lijun@webmail.hzau.edu.cn (J.L.); huangjunhong@webmail.hzau.edu.cn (J.H.); daimenghong@mail.hzau.edu.cn (M.D.); 2 MOA Laboratory for Risk Assessment of Quality and Safety of Livestock and Poultry Products, Huazhong Agricultural University, Wuhan 430070, China

**Keywords:** *E. coli*, olaquindox, resistance, sub-inhibitory concentration, transcriptome sequencing, whole genome sequencing

## Abstract

Quinoxaline1,4-di-*N*-oxides (QdNOs) are a class of important antibacterial drugs of veterinary use, of which the drug resistance mechanism has not yet been clearly explained. This study investigated the molecular mechanism of development of resistance in *Escherichia coli* (*E. coli*) under the pressure of sub-inhibitory concentration (sub-MIC) of olaquindox (OLA), a representative QdNOs drug. In vitro challenge of *E. coli* with 1/100× MIC to 1/2× MIC of OLA showed that the bacteria needed a longer time to develop resistance and could only achieve low to moderate levels of resistance as well as form weak biofilms. The transcriptomic and genomic profiles of the resistant *E. coli* induced by sub-MIC of OLA demonstrated that genes involved in tricarboxylic acid cycle, oxidation-reduction process, biofilm formation, and efflux pumps were up-regulated, while genes involved in DNA repair and outer membrane porin were down-regulated. Mutation rates were significantly increased in the sub-MIC OLA-treated bacteria and the mutated genes were mainly involved in the oxidation-reduction process, DNA repair, and replication. The SNPs were found in *degQ*, *ks71A*, *vgrG*, *bigA*, *cusA*, and *DR76_-_4702* genes, which were covered in both transcriptomic and genomic profiles. This study provides new insights into the resistance mechanism of QdNOs and increases the current data pertaining to the development of bacterial resistance under the stress of antibacterials at sub-MIC concentrations.

## 1. Introduction

In recent years, resistant bacteria infection is one of the most pressing concerns of global societies, which has substantially contributed to the clinic and economic burden of the healthcare system [1]. During the use of antibacterials, microorganisms are frequently exposed to drug concentrations below the minimal inhibitory concentration (MIC), which is called sub-inhibitory concentration (sub-MIC) [2]. The origins of sub-MIC concentration of the antibacterial environment are versatile. For example, the concentration of natural antibacterials produced by bacteria and fungi in the environment is often lower than the MIC of pathogens [3]. Furthermore, carcasses and excreta of animals that have used antibacterial drugs generate antibacterial gradients in the body and in the surrounding environment [4]. The poor therapeutic schedule of antibacterials treatment and preventive use of antibacterials, as well as the application of antibacterials as growth-promoters in livestock production are also important sources of sub-MIC concentrations [5]. These reservoirs of sub-MIC concentration of antibacterials involve humans, livestocks, crops, and aquaculture, as well as the related environment, which are intimately connected and provide favorable conditions for bacteria to develop antibacterial resistance.

Studies have shown that sub-MIC concentrations of antibacterial drugs can cause genetic and phenotypic changes in bacteria, including enrichment of the original drug-resistant bacteria, induction of new drug-resistant bacteria, formation of persisters, affecting bacterial biofilm formation, etc. [4]. Sub-MIC levels of certain types of antibacterials are known to trigger bacterial SOS response, resulting in the increase of mutation rates and lateral gene transfer [6]. It has also been suggested that sub-MIC levels of antibacterials may be more relevant to the problem of bacterial resistance than lethal concentrations of antibacterials [7,8,9]. One study evolved multiple independent lineages of wild-type *Escherichia coli (E. coli)* and *Salmonella typhimurium* strains under constant exposure of 1/10× MIC levels of ciprofloxacin and streptomycin, respectively, to assess the emergence of de novo resistance [10]. However, only a few studies address the development of bacterial resistance under long-term exposure of wide range of sub-MIC concentrations [11]. In addition, most of the researches on the mechanism of bacterial resistance induced by sub-MIC concentrations of antibacterials are on human drugs, while few studies are on veterinary drugs.

Quinoxaline1,4-di-*N*-oxides (QdNOs) have been widely used at sub-therapeutic levels to promote growth and improve effificiency of feed conversion in animal feeding for more than half a century [12]. Previous investigations have demonstrated that QdNOs can inhibit DNA synthesis and cause DNA damage [4,5]. It was suggested that bacterial reductase(s) are involved in the metabolic activation of QdNOs and some unstable intermediate products, likely free radicals that may attack DNA and induce DNA damage [4]. Olaquindox (OLA) is a classical member of QdNO family, which has a broad antimicrobial spectrum and can improve animal production performance [13,14]. However, it was demonstrated that the usage of OLA on pig farms increased the emergence of QdNOs resistance in fecal *E. coli* [15]. The OLA-resistant bacteria are often co-resistant to ampicillins, tetracyclines, and/or chloramphenicols [16]. The only known mechanism of QdNOs resistance is OqxAB efflux pump, a novel transmissible resistance-nodulation-division (RND) efflux pump, whose encoding genes, *oqxA* and *oqxB*, are generally located on chromosome or plasmid, conferring resistance to ampicillin, chloramphenicol, ciprofloxacin, and OLA in *E. coli* [17]. The *oqxAB-*located plasmid has a high frequency of transfer among enterobacterial pathogens, such as *S. Typhimurium*, *Klebsiella pneumoniae*, *Kluyvera* spp, *Enterobacter aerogenes*, etc. [18]. However, previous studies have found that there is no unique relationship between *oqxAB* gene and QdNOs resistance. OLA-resistant *E. coli* induced by OLA in vitro does not carry *oqxAB* gene, and one out of 21 OLA-resistant *E. coli* isolated from pigs did not contain the *oqxAB* gene [13]. From the human intestinal flora treated with another member of QdNOs, cyadox, only 26% (12 in 46) of QdNOs resistant *E. coli* contained *oqxAB* gene, and all resistant enterococci did not contain the *oqxAB* gene [19]. These data indicate that bacteria can obtain QdNOs resistance through other novel mechanism except the OqxAB-mediated QdNOs resistance. Previous studies have found that the reductive metabolic activity of quindoxin-induced *E. coli* mutants was reduced compared to the maternal bacteria [20]. Using suppression subtractive hybridization polymerase chain reaction (SSH PCR) subtraction, differentially expressed genes (DEGs) were selected in the QdNOs-resistant *E. coli* strains by OLA in vitro, and most of these genes were involved in the tolerance to oxidative stress [13]. However, the drawback of SSH PCR is that it can only select the up-regulated genes. Besides *oqxAB* genes, there is an urgent need to explore the novel mechanisms involved in the emergence of QdNOs resistance in bacteria.

This study aims to elucidate the dynamic phenotype changes and regulatory patterns of the development of bacterial resistance under the pressure of sub-MIC concentration of OLA, and to explore the new mechanism of the de novo QdNOs resistance, using whole genome and transcriptome sequencings. Clarifying the substantial effect of sub-MIC concentrations of QdNOs on the bacterial resistance will provide new insights for assessing the risk of antibacterial resistance caused by the low concentration of antibacterial agents.

## 2. Results

### 2.1. Phenotypic Changes of E. coli ATCC25922 Induced by Sub-MIC Of OLA In Vitro

#### 2.1.1. Resistance Development of *E. coli* ATCC25922 Exposed to Sub-MIC of OLA

**Figure 1:**
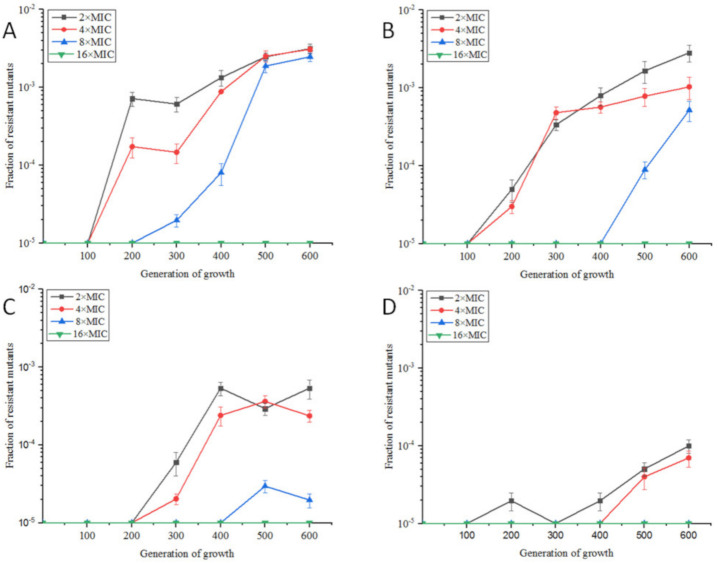
Resistance rates of *E. coli* ATCC25922 exposed to OLA at sub-MIC concentrations of 1/2× MIC (**A**), 1/4× MIC (**B**), 1/10× MIC (**C**) and 1/100× MIC (**D**).

Under aerobic conditions, the MIC value of OLA against *E. coli* ATCC25922 was 8 μg/mL. *E. coli* ATCC25922 was induced by sub-MIC of OLA in four dosage groups, 1/2× MIC(4 μg/mL), 1/4× MIC(2 μg/mL), 1/10× MIC(0.8 μg/mL), and 1/100× MIC(0.08 μg/mL). As shown in Figure 1, with the number of passages increased, the resistance rates gradually increased. From the 200th generation, 1/2× MIC and 1/4× MIC OLA-induced bacteria appeared colonies on plates containing 2, 4, and 8 times MIC of OLA, and the highest resistance rate was above 3‰ in the 600th generation. Bacteria induced by 1/10× MIC and 1/100× MIC OLA survived against 2× MIC and 4× MIC of OLA from the 300th and 500th generations, respectively, and the highest resistance rate was below 1‰ in the 600th generation. There were almost no drug-resistant colonies on the 16× MIC OLA plate for all the four groups, and 1/100× MIC OLA-induced group even had no resistant-colonies to 8× MIC OLA.

Differently, 1/2× MIC and 1/4× MIC Enrofloxacin (ENR)-induced *E. coli* appeared colonies on 2× MIC and 4× MIC ENR plates from 100 generations, and the resistance rates for 2× MIC ENR exceeded 20% by the 600th generation (Figure S1). Bacteria induced by 1/10× MIC and 1/100× MIC ENR survived against 2× MIC and 4× MIC of ENR from the 200th and 400th generations, respectively, and the resistance rate of the 1/10× MIC ENR-induced group to 2× MIC was more than 1% at the 500th generation, and the resistance rate of the 1/100× MIC ENR-induced group against 2× MIC reached 0.1% at the 600th generation. The resistance rates of the four groups in plates containing 8× MIC ENR at the 600th passage were 1.9%, 0.9%, 0.02%, and 0.003%, respectively. Except 1/100× MIC ENR group, the other three groups could induce resistant colonies against 16× MIC ENR at the 600th passage eventually.

#### 2.1.2. Effects of Sub-MIC of OLA on the Morphology and Biofilm Formation of *E. coli* ATCC25922

**Figure 2:**
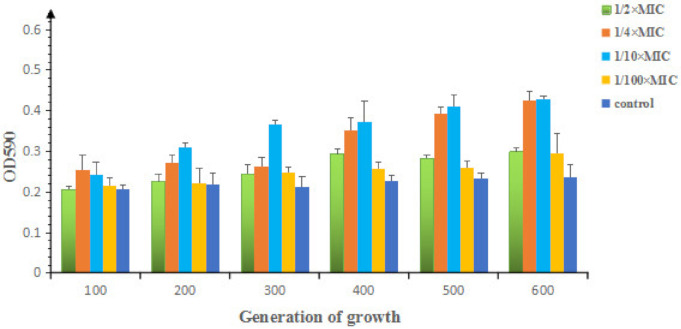
Biofilm formation of *E. coli* ATCC25922 induced by sub-MICs of OLA.

Scanning electron microscopy of 1/2× MIC OLA or ENR-induced bacteria showed that the sub-MIC of antibacterials had little effect on the growth or morphology of *E. coli* ATCC25922 (Figure S2). As shown in Figure 2, the biofilm formation ability of *E. coli* increased slightly with the increase of number of passages. Compared with control group, most of them belong to weak biofilm formation strains (OD value was 1 to 2 times that of the control group), and strong biofilm formation strains (OD value was 2 times larger than that of the control group) are 500th and 600th bacteria induced by 1/10× MIC and 600th bacteria induced by 1/4× MIC OLA.

### 2.2. Transcriptomic Profiles of E. coli ATCC25922 Induced by Sub-MIC of OLA

1/2× MIC and 1/10× MIC OLA*-*induced *E. coli* ATCC25922 which were resistant to 8× MIC OLA and the wild-type *E. coli* ATCC25922 were subjected to RNA sequencing respectively. Number of 20,857,965, 22,396,241 and 20,487,346 trimmed reads were obtained for the untreated, 1/2× MIC and 1/10× MIC OLA-treated bacteria, respectively (Table S1). The reads were mapped on the reference genome of *E. coli* ATCC25922 (NCBI accession No. GCF_000743255.1) (Supplementary Table S2). The closer the value of the correlation coefficient between samples is to 1, the more similar the expression patterns indicated. The correlation coefficients for gene expression pattern between biological triplicates of untreated, 1/10× MIC and 1/2× MIC OLA-treated bacteria were all ≥0.90, indicating that the correlation between triplicate samples in the same group is good. The square of the Pearson correlation coefficient (R^2^) varied from 0.80 to 0.97 in 1/2× MIC OLA-treated group and from 0.85 to 0.94 in 1/10× MIC OLA-treated samples, indicating good operational stability and reliability of sequencing library (Supplementary Figure S3). The functional annotation was performed based on the KEGG, NOG, GO, and NR databases. The annotation of unigenes in the Swiss-Prot, KO, eggNOG, GO, and NR databases accounted for 86.73%, 63.22%, 13.09%, 49.43%, and 99.76% of the total unigenes, respectively (Supplementary Table S3), indicating that the quality of transcriptome sequencing was good and the function of most genes was annotated. The RNAseq data were deposited in the NCBI Sequence Read Archive (SRA) database under the accession number of PRJNA525186.

DEGs between OLA-treated and untreated groups were analyzed to better understand the mechanism of bacterial resistance development against OLA (Supplementary Tables S4 and S5). Compared to the untreated group (group B), 156 genes were up-regulated and 130 were down-regulated in 1/2× MIC OLA treated group (group C). A comparison of gene expression between group B and 1/10× MIC treated group (group D) showed that 255 genes were differentially expressed, of which 101 genes were up-regulated and 154 genes were down-regulated. A total of 269 differentially expressed genes between the group C and group D with 85 genes up-regulated and 184 genes down-regulated. An investigation of the lists of DEGs by Venn diagram found that a number of the same genes were up-regulated or down-regulated in the OLA-treated groups compared with the untreated group (Supplementary Figure S4). There were 121 co-differentially expressed genes in the OLA-treated groups, 38 genes were up-regulated and 83 genes were down-regulated. Co-differentially expressed genes which have been annotated in the GO and KEGG databases, are shown in Table 1. Among them, three of the up-regulated genes are related to transcriptional regulators, fourteen genes are involved in the redox process of bacterial metabolism, six genes encode phosphoglucose transferase system (PTS) proteins, six genes are involved in sugar and amino acid metabolism, and six genes are associated with biofilm formation, ferrous iron transport, anti-adapter protein, formate C-acetyltransferase, sensor histidine kinase, and one uncharacterized protein, respectively. Only 21 down-regulated genes were annotated in the GO and KEGG databases. Nine genes are involved in DNA binding and repair, four genes encode the membrane component proteins, two genes encode chemotactic proteins, and two genes encode phage shock proteins. Other five genes encode pyrimidine utilization transport protein, putative electron transport protein, aminopeptidase N, toxin HigB-1 and a hypothetical protein, respectively.

**Figure 3:**
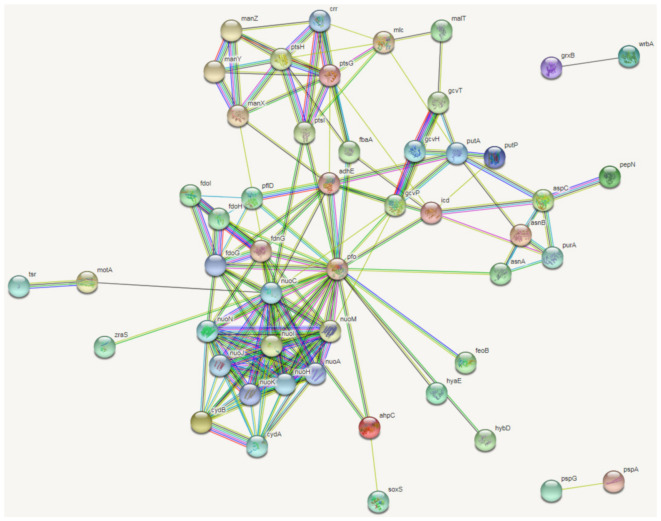
Protein–protein interactions of *E. coli* ATCC25922 induced by sub-MICs of OLA generated following analysis of significantly different peptides input into the STRING database. The figure shows the protein–protein interactions networks that resulted from co-differentially expressed genes. “
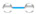
” represent known interactions from curated databases, “
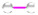
” represent known interactions have been experimentally determined; “
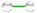
” represent predicted interactions of gene neighborhood, “
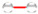
” represent predicted interactions of gene fusions, “
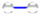
” represent predicted interactions of protein homology. “
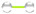
” represent interactions of gene textmining, “
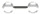
” represent predicted interactions of gene co-expression, “
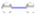
” represent interactions of gene co-occurrence.

The proteins corresponding to the 121 co-differentially expressed genes were matched with the proteins of *Escherichia coli* K12 MG1655, and then a cluster analysis of protein interactions was performed with 15 significant KEGG metabolic pathways obtained in the STRING database (Figure 3). They are phosphotransferase system (PTS), metabolic pathways, alanine, aspartate and glutamate metabolism, carbon metabolism, biosynthesis of antibiotics, microbial metabolism in diverse environments, biosynthesis of secondary metabolites, glyoxylate and dicarboxylate metabolism, amino sugar and nucleotide sugar metabolism, fructose and mannose metabolism, glycolysis/gluconeogenesis, methane metabolism, tyrosine metabolism, butanoate metabolism, oxidative phosphorylation. In this network, oxidoreductases, such as Pfo, AdhE, and NuoM were shown to play a role in the important processes of pyruvate-ferredoxin/flavodoxin redox, DNA replication, and NADH-quinone redox. Because these proteins interact with more proteins, they are the key nodes of the protein interaction network.

Compared with the reference genome sequence of *E. coli* ATCC25922 (NCBI accession number GCF_000743255.1), RNA-seq results showed that the number of SNPs in the genome were 193, 120, and 122 from the three duplicates of 1/10× MIC OLA treated group, 128, 146, and 129 from the three duplicates of 1/2× MIC OLA treated group, and 86, 92, and 85 from the three duplicates of untreated group, respectively. RNA-seq results also showed that the number of indel sites in the genome was 30, 21, and 22 from the three duplicates of 1/10× MIC OLA treated group, 19, 24, and 18 from the three duplicates of 1/2× MIC OLA treated group, and 13, 18, and 12 from the three duplicates of untreated group, respectively. This result demonstrated that the number of mutations and insertion/deletion sites in the OLA treated groups was significantly increased. Among these SNPs, 23 SNP sites would result in a protein sequence change, 15 non-synonymous mutations, and eight single-point deletions (Table 2). Genes with these mutations encode regulatory proteins of transcription, nucleotide pyrophosphatase family proteins, efflux system membrane proteins, etc. The SNPs were compared with the CARD resistance database, and the results showed that there were no mutations of the known drug resistance genes.

### 2.3. Genomic Profiles of E. coli ATCC25922 Induced by Sub-MIC Of OLA

**Figure 4:**
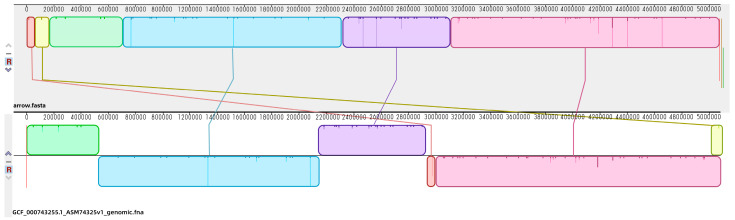
Genome comparisons of the selective *E. coli* genome. The alignment between OLA-treated *E. coli* ATCC25922 genome (above) and reference *E. coli* ATCC25922 (NCBI accession No. GCF_000743255.1) (below) is displayed as one horizontal panel per input genome sequence. Colored blocks in the first genome are connected by lines to similarly colored blocks in the second genomes. These lines indicate which regions in each genome are homologous. When a block lies above the center line the aligned region is in the forward orientation relative to the first genome sequence. Blocks below the center line indicate regions that align in the reverse complement orientation. Inside each block Mauve draws a similarity profile of the genome sequence. The height of the similarity profile corresponds to the average level of conservation in that region of the genome sequence.

The genome of *E. coli* ATCC25922 induced by 1/2× MIC OLA and resistant to 8× MIC OLA showed a total bases of 529,982,985 bp and a number of subreads of 64,908, with average subreads length of 8165 bp. Using the Canu software, the genome was assembled and showed a 5,158,951 bp length (Supplementary Figure S5), which contained 5384 predicted genes with an average G+C content of 50.49%. The protein sequences predicted by Glimmer software were extracted, and the length of the protein sequences were statistically plotted (Supplementary Figure S6). There were 4045 and 3626 predicted genes annotated in COG (Supplementary Figure S7) and GO database, respectively (Supplementary Figure S8). Also, 3181 predicted genes were involved in 29 KEGG pathways which were classified into “Metabolism” (12 out of 29), “Genetic Information Processing” (four out of 29), “Cellular Processes” (four out of 29), “Environmental Information Processing” (two out of 29), and “Organismal Systems”(seven out of 29) (Supplementary Figure S9). The genomic sequencing data were deposited in the NCBI Sequence Read Archive (SRA) database under the accession number of PRJNA525188. Using the genomic multiple alignment tool Mauve, the genome of the OLA resistant *E. coli* was compared with the reference genome of *E. coli* ATCC25922 (NCBI accession No. GCF_000743255.1). There are two gene fragments of shifting and one gene fragment of deletion (Figure 4).

Using MUMmer (v3.23), the genome of the OLA-resistant bacteria was compared with the reference genome (NCBI accession No. GCF_000743255.1), and 715 SNP sites were obtained, among which 270 SNP sites involving 41 genes existed in the exon region, causing non-synonymous mutations or codon termination. In the meantime, 82 indel sites were obtained, among which 27 indel sites existed in the exon region (Table 3). There were 113 SNP sites and 19 indel sites associated with the DNA replication fork binding protein CrfC, which plays important functions such as cell replication, recombination, and repair. Only three SNP sites and one indel locus were involved in DNA binding, transcriptional regulation, and recombination genes. There was an SNP site observed in drug/metabolite transporter gene *yddG* and a SNP site efflux gene *acrB*. There were four SNP sites and one non-frameshift deletion found in NAD^+^ oxidoreductase gene. These results indicated that the bacterial resistance to QdNO drugs could be related to NAD^+^ redox and efflux pump. Comparing the SNPs between the transcriptomic sequencing (Table 2) and genomic sequencing (Table 3), six identical SNP sites, *degQ*, *ks71A*, *vgrG*, *bigA*, *cusA*, and *DR76_-_4702*, were found. *vgrG* and *bigA* have different mutation sites, and the other 4 genes have only one mutation site in both of the sequencing profiles. Other SNPs included amino acid metabolism, integral component of membrane, ion transport, and nucleic acid metabolism.

### 2.4. The Susceptibilities of Sub-MIC OLA-Induced Resistants against Other Antimicrobial Agents

The MICs for the wildtype, 1/2× MIC and 1/10× MIC OLA induced 8× MIC OLA resistant *E. coli* ATCC25922, against other antimicrobial agents were determined using the broth micro-dilution method [17]. As shown in Table 4, 1/2× MIC OLA treated group and1/10× MIC OLA treated group had reduced sensitivity to ampicillin, florfenicol, compound sulfamethoxazole (Sulfamethoxazole +Trimethoprim), and acetylquine. There was no change in the sensitivity of resistant bacteria to augmentin (Amoxicillin + Clavulanic acid), tetracycline, spectinomycin, ceftiofur, ceftazidime, enrofloxacin, ofloxacin, meropenem, and apramycin. However, the sensitivity of resistant bacteria to colistin has increased.

## 3. Discussion

### 3.1. Phenotype Characteristics of Sub-MIC Antimicrobial-Induced Bacterial Resistance

The previous study has demonstrated that two-fold ascended concentrations of OLA(from 1/2× MIC to 4× MIC) can contribute to the selection and maintenance of OLA-resistant bacteria against 16× MIC OLA in 20 days [13]. However, in this study, under sub-MIC concentrations of OLA (from 1/100× MIC to 1/2× MIC), bacteria took a longer time (60 days) to enrich resistants, with maximum resistant level of only 8× MIC (Figure 1). The step-wise selection by an antibacterial at concentration above MIC will enrich the existing resistant sub-population that acquire resistance by mutation or horizontal gene transfer, while sub-MIC selection can also drive the emergence of de novo resistant mutants [21], by increasing the mutation rate or stimulating the horizontal gene transfer in susceptive bacteria which do not die in sub-MIC concentrations [22]. Moreover, for the resistant mutants that already exist in the population, the sub-MIC concentration would enrich those with less fitness cost than the susceptive counterpart [10]. So, the exposure level of drug in clinical settings will influence the speed and the level of bacterial resistance development.

It has been demonstrated that stress responses gene *soxS* and type I fimbriae gene (*fimG*), metabolism gene (*metK*) were differentially expressed in all *E. coli* biofilm studies [23]. *bssR*, encoding a biofilm regulator, was discovered for the induction of biofilms in *E. coli* [24]. In this study, *soxS* and *bssR* as well as many carbon catabolism related genes were up-regulated in the OLA induced resistant bacteria (Table 1). Especially, the *bssR* gene was up-regulated by 2 times in the 1/2× MIC treated group and five times in the 1/10× MIC group, which was consistent with the amount of biofilm formation (Figure 2).

A phenomenon termed collateral sensitivity, whereby resistance to one antimicrobial simultaneously increases the susceptibility to another, was reported [25]. Collateral susceptibilities of mutants resistant to relevant antimicrobials against 16 antibiotics have been demonstrated, and it was shown that resistance mechanisms, in particular efflux-related mutations, as well as the relative fitness of resistant strains, are principal contributors to collateral responses [26]. Csaba et al also reported that various mechanisms of resistance, including target mutations and those mutations affecting drug uptake and efflux, are prone to induce collateral sensitivity [27]. Our data showed that resistance to OLA resulted in collateral responses to mequindox, ampicillin, compound sulfamethoxazole, florfenicol. However, the susceptibility to colistin of the OLA resistants decreased. The mechanism of action of mequindox is targeted to DNA and compound sulfamethoxazole affect the synthesis of folate. Florfenicol acts on the 50S ribosome of bacteria and amoxicillin inhibits the formation of bacterial cell walls. These results suggest that collateral susceptibility may be associated with antimicrobial resistance mechanism. The above results show that evolution of resistance towards a given antimicrobial agent frequently increases resistance to several other drugs. but it has remained unclear how frequently evolution of resistance increases sensitivity to other drugs. Cross-resistance profiles may be strongly correlated with drug resistance trajectories and the antibacterial mechanism of antibiotics. In addition, fitness costs may slow the evolution of the collateral responses.

### 3.2. Molecular Mechanism of Sub-MIC of QdNO-Induced Resistance

The PTS system located in the inner membrane is a multiprotein phosphorylation system that couples the transport of carbohydrates across the cytoplasmic membrane with their simultaneous phosphorylation. The global repressor Mlc regulates the expression of phosphoenolpyruvate: sugar phosphotransferase system (PTS) operon which is related to the uptake and utilization of sugars. It has been reported that *mlc* inhibits *manXYZ*, *malT*, *ptsG*, and *pts* operon, encoding enzyme II of the mannose PTS, the activator of maltose operon [28], enzyme IICB of the glucose PTS, and other PTS proteins, respectively. Our results showed that mutation appears among the *mlc* sequence, so the mlc may lose the ability of inhibiting *manXYZ*, *malT*, *ptsG,* and *pts* operon. MalT is a transcriptional regulator of the maltose regulon, which is involved in regulating the transport of maltose [29]. Under sub-MIC of OLA, it was shown that the expression of PTS system genes including *mlc*, *manX*, *manY*, *manZ*, *ptsG*, *ptsH*, *ptsI,* and non-PTS system gene *malT* were up-regulated. It can be speculated that the expression of the mannose phosphate transfer system is up-regulated, and the maltose transport system in the non-PTS is also more active. This indicates an increase in the uptake of carbon sources in resistant bacteria. Compared to the wild-type *E. coli* ATCC25922, some genes involved in glycolysis were up-regulated in the resistant bacteria induced by 1/2× MIC OLA, including *fbaA* (fructose-bisphosphate aldolase), *fbaB* (fructose-bisphosphate aldolase), and *gap*(glyceraldehyde 3-phosphate dehydrogenase). In 1/10× MIC OLA induced bacteria, there are also some genes involved in glycolysis up-regulated, including *pgk* (phosphoglycerate kinase), *fbaA*, *pfkB* (6-phosphofructokinase), *ppsA* (pyruvate, water dikinase), and *gldA* (glycerol dehydrogenase). Genes involved in pyruvate metabolic pathways, such as *pfo (nifJ)*, were up-regulated. So, the pyruvate metabolism was up-regulated and produces more acetyl-CoA for the energy system. Moreover, many other metabolic pathways, such as glycine, histidine, and aspartate metabolism, were involved in the response to OLA as well. Genes involved in amino acid metabolism were also up-regulated, such as *gcvP*, *zraS,* and *asnB*. The metabolism of the major carbon and nitrogen substrates are afforded by multiple specific uptake systems and pathways. The genes involved in these pathways are controlled by both global and specific transcriptional regulators that may directly or indirectly sense key metabolites that are generated in the central carbon and nitrogen metabolic pathways [30]. Our study indicates that the metabolism of sugars and amino acids is enhanced in *E. coli* resistant to OLA, probably to remove oxidative damage and accelerate the synthesis of new substances to maintain the normal physiological function of the cells.

The *adhE* (aldehyde dehydrogenase) and *icdA* (isocitrate dehydrogenase) were up-regulated in this study, so the yield of NADPH increased. In the meantime, the over-expression of *adhE* increased the yield of acetaldehyde, thus reducing the amount of acetyl-CoA in the TCA cycle, resulting in the decrease of NADH/NAD ratio [31], playing a protective role against oxidative stress [32]. NifJ, a pyruvate ferredoxin (flavodoxin) oxidoreductase, catalyzes the oxidation of pyruvate to acetyl-coenzyme A and carbon dioxide in bacteria. It was revealed that NifJ can balance NADH-mediated process by regulating the NADH/NAD ratio [33], so the overexpressed of the *nifJ* gene also cause the reduction of NADH/NAD ratio. *wrbA* is a tryptophan repressor-binding protein that binds one molecule of flavin mononucleotide, which is involved in oxidative defense [34]. It appears to be a big electron acceptor pool to accommodate the electron of the electron donor, NADH or benzoquinone [35]. The CydAB quinol oxidases catalyze the reduction of oxygen by ubiquinol. *cydB* gene encodes a subunit of CydAB quinol oxidases, and the accumulation of NADH was prevented when the *cydB* mutated [36]. CydAB also play an important role in protection against oxidative stress by reducing H_2_O_2_ content [37]. *nuoM* and *nuoI* encode NADH:ubiquinone oxidoreductase, whose hydrophilic domain catalyzes transferring two electrons from NADH to quinone [38]. In the resistant bacteria induced by the sub-MIC of OLA in our study, *adhE*, *nifJ*, *wrbA*, *cydB*, *nuoM,* and *nuoI* were significantly up-regulated (Table 1). It was reported that QdNOs was metabolized by a reductase to produce free radicals in bacteria and this process also involved electron transfer [39]. Therefore, reducing the accumulation of NADH by decreasing the NADH/NAD ratio and increasing the NADPH/NADP ratio might be one of the mechanisms of QdNO resistance.

MarA, SoxS and Rob are three homologous transcriptional regulators of *E. coli*. These homologous regulators are part of multiple regulatory mechanisms necessary for the adaptive response, including changing pH, the presence of antibiotics, oxidative stressors, and organic solvents, all of which threaten survival [40]. The *soxRS* regulon is involved in oxidative stress response and also responsible for the resistance of *E. coli* to antibacterials [41,42]. Only the upregulation of SoxS was found in our study, but not MarA and Rob. The possible reason for this is that cross talk between the mar and sox systems is limited under physiological conditions. Previous study showed that the expression of SoxS is not expected to activate marRAB transcription in the absence of salicylate, when MarR still represses the promoter [42]. A previous study also showed that SoxR is regulated by NADPH-dependent reductases, an increased NADPH demand of the cell counteracts SoxR reduction and increases soxS expression [43]. Several genes encoding NADPH-dependent reductases were up-regulated, such as nuoG, nuoH, and nuoI. So, the up-regulation of these genes may cause the up-regulation of soxS. Previous study showed that the alkyl hydroperoxide reductase (AhpC) has the function of scavenging ROS and peroxides in variety of aerobic and anaerobic bacteria [44,45]. Previous research has suggested that the knockout of *grxB* gene, which encodes glutaredoxins ubiquitous protein, cause lower levels of oxidoreductase activity, resulting in the increased sensitivity to oxidative stress in *E. coli* [46]. Overproduction of the electron transfer subunits of formate dehydrogenases, encoded by the *fdoH*, *fdoI*, and *fdnG*, can enhance oxidative stress tolerance [47,48]. In our study, *soxS*, *ahpC*, *grxB*, *fdoH*, *fdoI,* and *fdnG* gene were up-regulated. Our previous study shows that QdNO-induced DNA damage is related to the yield of free radicals, and free radical scavengers can alleviate the bactericidal activity of cyadox [49]. Therefore, free radical scavenging and oxidative stress tolerance may be one of the mechanisms of QdNOs resistance.

*umuC* encodes polymerase V, a low-fidelity DNA polymerase, which has the ability to trigger a repair pathway during the SOS response to DNA damage [50]. The RusA protein is a DNA structure-specific endonuclease that resolves holliday junction intermediates formed during DNA repair [51]. RutG, a broad-specificity pyrimidine permease, is predicted to be a pyrimidine transporter [52]. Previous study demonstrated that the genes involved in DNA metabolic process and SOS response were downregulated, including *dinB*, *umuC*, *recA*, etc. [53]. In this study, DNA damage repair genes, *umuC*, *rusA*, and *rutG* were down-regulated. The resistant bacteria may resist DNA damage, but the susceptive bacteria can be damaged by antibiotics. Therefore, the expression of genes related to DNA repair in drug-resistant bacteria is down-regulated.

A total of 50 non-synonymous SNPs and indels were observed between the genomes of 8× MIC OLA resistant bacteria and the reference strain (NCBI accession No. GCF_000743255.1). IraD is a factor aiding the survival of proliferating *E. coli* cells against multiple forms of DNA damage, including oxidative stress [54]. CdiA secretion protein provides immunity and inhibits the bacterial growth by a significant down-regulation of metabolic parameters, such as aerobic respiration, steady-state ATP levels, etc. [55]. A previous study suggests that the Type VI Secretion System core component VgrG contributes to the antimicrobial resistance in *A*. *baumannii* ATCC19606 [56]. The gene *ydeE* encodes a multidrug-efflux transporter of *E. coli* which confers resistance to dipeptides [57]. Previous studies reported that the cytotoxicity of bactericidal antibacterials predominantly results from lethal double-strand DNA breaks caused by incomplete repair of 8-oxo-deoxyguanosine (8-oxo-dGTPase), which is encoded by *mutT* gene to prevent incorporation of base analogs into DNA [58]. *iraD*, *cdiA*, *vgrG*, *ydeE,* and *mutT* genes involved in DNA damage, bacteria growth and antimicrobial resistance were mutated in our study, which may be related to the resistance of QdNOs. Previous study demonstrates that CrfC protein, distributed in the nucleoid poles, helps sustain the colocalization of nascent DNA regions of sister replisomes and promote chromosome equipartitioning [59]. So, the CrfC might be involved in reactions necessary for chromosome stability and protect DNA damage.

Comparing the SNPs in the transcriptomic data (Table 2) with those in the genomic data (Table 3), six identical SNP genes were found. They are *degQ*, *ks71A*, *vgrG*, *bigA*, *cusA,* and *DR76-4702*. DegQ, a HtrA protease of the *E. coli*, is involved in the biosynthesis of outer membrane proteins [60,61]. *ks71A* encodes the fimbrial subunits of *E. coli* Ks71A, which is a hypothetical protein [62]. *vgrG is a* valine–glycine repeat G gene, previous study showed that the deletion of the vgrG gene, increased antimicrobial resistance to ampicillin/sulbactam, but reduced resistance to chloramphenicol [56]. The vgrG gene was mutated in the genome profiles. So, the vgrG gene might be related to OLA resistance. *bigA* encodes a putative surface-exposed virulence proteinin *E. coli*, and it also has been reported that the BigA protein acted as an adhesin protein of *Brucella* that mediates adhesion to epithelial cells [63]. CusA is an inner membrane proton-substrate carrier, responsible for transferring Cu(I) and Ag(I) ions [64]. The *DR76-4702* gene has not been named, encoding a putative integral component of membrane. The outer membrane (OM) of Gram-negative bacteria performs the crucial role of providing an extra layer of protection to the organism, so the mutation of these genes provided a formidable barrier for bacteria to sustaining life.

**Figure 5:**
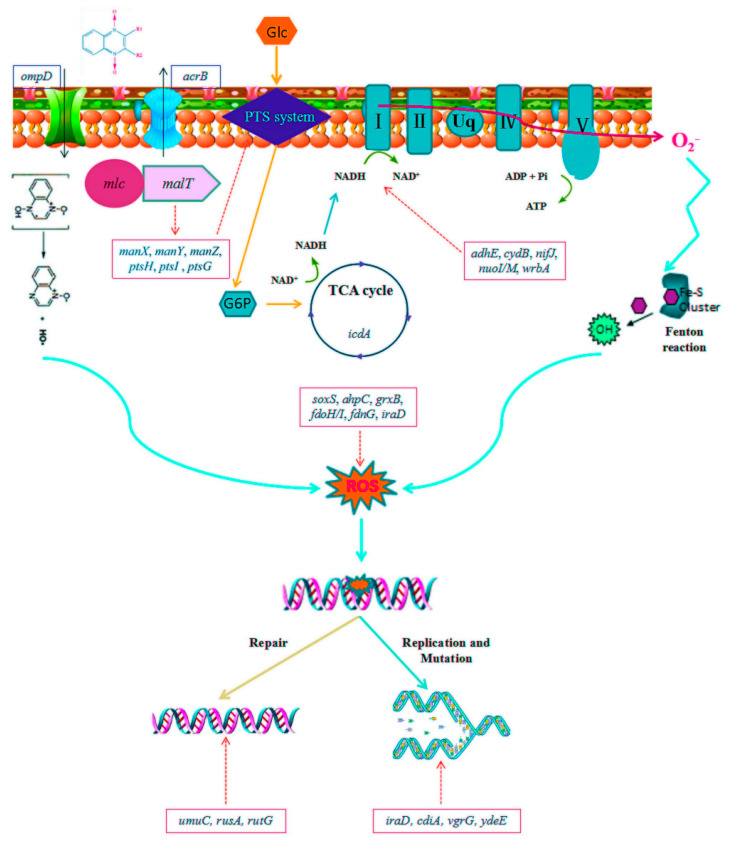
The hyperthetical resistance mechanism involved in the development of QdNOs resistance. The decrease in the expression of the outer membrane porin gene ompD leads to the loss of OmpD protein, which reduces the intake of olaquindox. The up-regulation of multidrug resistance efflux pump gene acrB increases olaquindox efflux. The PTS systerm initiate a metabolic feedback dependent on tricarboxylic acid (TCA) cycle, stimulate the oxidation of NADH through the electron transport chain, promote superoxide formation, damage the structure of Fe-S clusters, lead to the formation of hydroxyl radicals and finally damage DNA; The Changes in the expression of genes umuC, rusA, and rutG trigger DNA repair; Changes in the expression of genes such as iraD, cdtA, vgrG, and ydeE cause gene mutations and affect DNA replication.

A previous study has showed that the mechanism of an oxidative damage cellular death pathway involving the tricarboxylic acid cycle, a transient depletion of NADH, destabilization of iron-sulfur clusters, and stimulation of the Fenton reaction [65]. It was demonstrated that QdNOs genes and proteins involved in the bacterial metabolism, cellular structure maintenance, resistance and virulence were found to be changed, accompanying the SOS response and oxidative stress induced [49]. A previous study revealed that *E. coli* adapt the stress generated by QdNOs or develop specific QdNOs-resistance by the activation of antioxidative agent biosynthesis, protein biosynthesis, glycolysis, and oxidative phosphorylation [13]. Based on the mechanism of cell death induced by QdNOs and oxidative stress-mediated mode of action proposed for QdNOs in the above literature, it is supposed that the QdNOs resistance mechanism involved in outer membrane porin, efflux pump, the tricarboxylic acid (TCA) cycle, the oxidation of NADH, damage DNA repair and DNA replication and mutation of *E.coli* (Figure 5). The study only elaborated the resistance mechanism of *E. coli* to OLA at sub-inhibitory concentrations of OLA, and could not fully cover all the resistance mechanisms of OLA under concentrations above MIC. This study only studied the OLA resistance mechanism of QdNOs, which may not represent the resistance mechanism of all QdNOs. Therefore, it is necessary to study the resistance mechanism of other QdNOs.

## 4. Materials and Methods

### 4.1. Drugs, Bacteria and Growth Conditions

OLA (purity of 99.8%) was purchased from National Institutes for Food and Drug Control (Beijing, China). Enrofloxacin (ENR) was bought from China Institute of Pharmaceutical and Biological Products Inspection (Beijing, China). The standard solutions of drugs were prepared according to the manufacturer’s instruction at concentration of 1280 mg/L. *E. coli* ATCC25922 was purchased from China Center for Type Culture Collection (CCTCC). Mueller–Hinton broth, Mueller–Hinton agar (Mueller–Hinton broth supplemented with 1.5% agar) and Luria–Bertani (LB) agar was bought from HOPEBIO (Tsingtao, China). All other chemical reagents were of analytical grade.

### 4.2. Antimicrobial Susceptibility Testing

The MICs of antibacterials for wild-type and OLA-resistant *E. coli* ATCC25922 were determined using the broth micro-dilution method, according to the guidelines of the Clinical and Laboratory Standards Institute (CLSI) [66]. Specifically, 0.1 mL of two-fold serial dilutions of drugs were prepared and inoculated with 0.1 mL of bacterial overnight culture diluted into 5×10^5^ CFU/mL. The tubes were incubated at 37 °C with shaking for 16–18 hours, the OLA cultures protected from light to avoid degradation of the antibiotic. The MIC was defined as the lowest concentration of an antimicrobial able to completely inhibit the microbial growth, as described in the CLSI protocols.

### 4.3. In Vitro Selection of Resistant Bacteria under Sub-Inhibitory Concentrations of OLA

To select for de novo generated resistant mutants at sub-MICs of OLA, a single colony of *E. coli* ATCC25922 was transferred into 5 mL of Mueller–Hinton broth and incubated at 37 °C overnight. Fresh cultures of bacteria were serially passaged by 1000-fold dilution in 5 mL batch cultures every 24 hours for 600 generations (60 passages, 10 generations per passage) in Mueller-Hinton medium containing OLA at concentrations of 1/2× MIC (4 μg/mL), 1/4× MIC (2 μg/mL), 1/10× MIC (0.8 μg/mL), and 1/100× MIC (0.08 μg/mL) or ENR at concentrations of 1/2× MIC (0.0075 μg/mL), 1/4× MIC (0.00375 μg/mL), 1/10× MIC (0.0015 μg/mL), and 1/100× MIC (0.00015 μg/mL) respectively, and the untreated *E. coli* culture was used as control. The liquid cultures were grown at 37 °C under aerobic conditions without shaking [10]. For every 100 generations of the bacteria culture, the number of colonies was counted, and the percentage of resistant cells in each culture was monitored by plating approximately 10^5^ cells onto LB agar containing different concentrations of OLA or ENR as control (2× MIC, 4× MIC, 8× MIC, and 16× MIC). A subset of these cells was restreaked on the same antibiotic concentration to confirm that they were resistants.

### 4.4. Bacterial Morphology Observation and Biofilm Detection

To investigate the morphological changes of bacteria under sub-MIC of antimicrobial drugs, *E. coli* ATCC25922 induced by 1/2× MIC OLA and 1/2× MIC ENR as well as untreated group were observed by scanning electron microscopy. Immobilization, embedding, sectioning, staining and observation of bacterial samples were carried out as previously described [67]. Bacteria (about 10^6^ CFU/mL) were harvested by centrifugation, washed twice with 0.1 M PBS (pH 7.4), and fixed in 0.2% glutaraldehyde in 0.1 M cacodylate buffer at 4 °C for 4 h, and then fixed in the perfluorocarbon containing 1% osmium tetroxide for 1 h. After three rinses in pure perfluorocarbon, the samples were dehydrated, embedded, sectioned, stained, and imaged with a TEM (H-7650, Japan) as previously described [68].

The method of biofilm formation detection of bacteria at various stages induced by 1/2× MIC, 1/4× MIC, 1/10× MIC, and 1/100× MIC of OLA was as follows [69]. After overnight incubation to logarithmic growth phase, the cultures were diluted to 0.5 McFarland standards in broth medium, respectively. A volume of 200 μL bacterial cultures was transferred to each well of a 96-well plate, and bacterial biofilms were formed followed by incubation at 37°C for 24 h with no movement. After discarding the medium and rinsing the wells of the 96-well plate 3 times with sterile PBS solution, the biofilms were placed in a 0.1% (wt/vol) crystal violet solution for 15 min, rinsed again, and dried for several hours. To solubilize the adsorbed crystal violet, wells with stained biofilms were incubated in 30% acetic acid for 15 min. The absorbance was read at 590 nm on a microplate reader (Thermo Fisher Scientific, Shanghai, China).

### 4.5. RNA Sequencing Analysis

#### 4.5.1. RNA Extraction, Cdna Library Construction and Sequencing

The total RNA of resistant *E. coli* ATCC 25922 induced by 1/2× MIC and 1/10× MIC OLA and exhibiting a MIC of 64 μg/mL (equal to 8× MIC of wild-type susceptive strain) was extracted using a bacterial RNAprep Pure Plant Kit (Tiangen, Beijing, China), following the manufacturer’s instructions. The concentration and purity of total RNA were measured on an Agilent 2100 Bioanalyzer (Agilent Technologies, Santa Clara, USA). Equal amounts of the total RNA from three replicates of untreated group (Group B), 1/2× MIC OLA-treated group (Group C) and 1/10× MIC OLA-treated group (Group D) were pooled for RNA-Seq library construction. The mRNA was enriched from qualified total RNA using Oligo(dT) magnetic beads and fragmented chemically into small pieces at high temperature. Then, the first cDNA strand was synthesized by using random hexamers for reverse transcription with cleaved RNA fragments serving as templates, followed by second-strand cDNA synthesis using DNA polymerase I and RNase H. The double-strand cDNA then went through purification, end repair process, the addition of a single “A” base and ligation of the adapters, and was finally enriched by PCR implication to be ready for sequencing via Illumina HiSeq™ 2000 (Personalbio technology Co. Ltd., Shanghai, China).

#### 4.5.2. Transcriptome Assembly and Gene Functions Annotation

A total of 39.0 GB raw data was obtained from nine samples via Illumina system. Clean reads were obtained after removing adapter sequences, filtering low-quality reads, and removing reads with an N ratio of greater than 5% from the raw reads. Each sample had more than 89.67% of clean data, with Q30 values greater than 90.03%. After adaptor sequences, ambiguous reads, and low-quality reads were removed, the sequence data were assembled with Trinity software [70]. RNA sequence reads were independently aligned with the genome sequence of *E. coli* ATCC 25922 (NCBI accession number GCF_000743255.1). Gene function was annotated based on the following databases: Nr (NCBI non-redundant protein sequences); Nt (NCBI non-redundant nucleotide sequences); Pfam (Protein family); COG (Clusters of Orthologous Groups of proteins); Swiss-Prot (A manually annotated and reviewed protein sequence database); KO (KEGG Ortholog database); and GO (Gene Ontology). Principal component analysis was performed using the R statistical computing package [71]. Gene ontology analysis was performed by Blast2GO through searching the above databases [72]. The GO terms assign biological process, molecular function, and cellular component to the query sequences. KEGG analysis was performed using the online KEGG Automatic Annotation Server (KAAS; http://www.genome.jp/kegg/kaas) to obtain an overview of gene pathways networks.

#### 4.5.3. Detection of DEGs

Gene expression was analyzed by DESeq (version 1.18.0). The screening conditions for DEGs included expression fold difference of log2|fold change| > 1 and significance of *p*-value <0.05.

#### 4.5.4. Identification of Antimicrobial Resistant Genes

The set of resistance-associated genes was downloaded from the Antibiotic Resistance Genes Database (http://ardb.cbcb.umd.edu/). DEGs were annotated using the tool retrieved from the same web site, which was performed based on a homology search by BLASTp. The cutoff parameters used in this study were E-value <1e^−5^ and nucleotide sequence identity >50 %.

### 4.6. Genomic Sequencing Analysis 

#### 4.6.1. DNA Preparation, DNA Library Construction and Sequencing

Genomic DNA of resistant *E. coli* ATCC 25922 induced by 1/2× MIC of OLA and exhibiting a MIC of 64 μg/mL(equal to 8× MIC for wild-type susceptive strain) was extracted using a bacterial genomic DNA purification kit (Tiangen Biotech Co., Ltd., Beijing, China) according to the manufacturer’s instructions. DNA was quantified using a NanoDrop ND-1000 spectrophotometer (NanoDrop Technologies, Wilmington, DE, USA). The isolated DNA was stored at –80 °C until use. Whole-genome sequencing was carried out using the PacBio Sequel platform (Frasergen Co. Ltd., Wuhan, China). A quantity of 5 μg input genomic DNA was used for 10 kb fragment library preparation. DNA was sheared with g-TUBE1 microcentrifuge tubes (Covaris, Woburn, MA) and SMRTbell libraries prepared with DNA Template Kit 2.0 (Pacific Biosciences, Menlo Park, CA). Sequencing primers were annealed to the SMRTbell template and samples were sequenced using C3 chemistry, Polymerase (version 5), SMRTAnalysis software (version 2.3.0), and a single SMRT cell (Pacific Biosciences, Menlo Park, CA). Raw read quality and average reference consensus were determined using SMRT Analysis software (version 2.3.0).

#### 4.6.2. Genome Assembly and Annotation

Filtered reads were obtained from PacBio raw data using SMRT Analysis and were assembled by applying the Canu protocol [73]. The assembled genome sequence was annotated by the Institute for Genomic Research database, using the programs Glimmer (version 3.02) for the identification of protein-coding genes [74], tRNAscan-SE for the identification of tRNA genes [75], and RNAmmer for the identification of rRNA genes [76]. The non-coding RNA were identified using the Rfam (version 12.0) and Infernal [77]. Short tandem repeat and simple sequence repeats were identified by trf407b.linux [78], and clustered regularly interspaced short palindromic repeats (CRISPRs) were identified using CRISPRs in Environmental Datasets [79].

### 4.7. Statistical Analysis

All statistical analyses were performed using GraphPadPrism software (GraphPad Software Inc., LaJolla, CA, USA) with all data represented as the mean ± standard deviation (SD) from at least three independent experiments. Data analyses were performed using the *t*-test or one-way analysis of variance (ANOVA), and a *p*-value less than 0.05 was considered statistically significant.

## 5. Conclusions

Our data show the evolution of resistance of *E. coli* de novo selected by sub-MIC concentrations of OLA, and provide a reference predicting collateral sensitivity informed therapy. QdNOs are redox-activated, hypoxia-selective DNA-cleaving agents [80]. The oxidative DNA-damaging effects of QdNOs will stimulate the SOS response, oxidative stress, and other protection strategies in bacteria. Combined with the previous study of the QdNOs resistance mechanisms [20], we further elaborated the molecular mechanism of QdNOs resistance under sub-MIC. The intake of QdNOs was first reduced by adjusting the expression of membrane porin and efflux pumps and mutations in efflux pump genes, mainly including *ompD* and *acrB*. When the QdNOs enters the bacterial cell, it stimulates the bacteria to feedback by regulating the electron transport chains, resulting in the production and consumption of NADH to reduce the production of free radicals. The main genes involved in this process are *soxS*, *malT*, *nouM*, *nouN,* and *icdA*. When an oxidative stress reaction occurs, the free radicals were cleared by regulating the expression of certain redox genes and genes involved in DNA repair, involving genes of *soxS*, *umuC*, *iraD*, *ahpC*, *wrbA,* and *rusA*. The bacteria cell division was halted when exposed to CYA [25], so the mutations in the *crfC* gene, which is involved in bacterial replication, may be another key gene of QdNOs resistance. The deeper molecular mechanism involved in the origin of resistance development selected by QdNOs should be explored. Further studies are required to investigate the signaling pathway of the origin of the QdNOs resistance. Targeting the key molecular actors involved in the gene expression and inhibiting the mutagenic pathways of the emergence of resistance could be a key strategy to slow down the emergence of antibacterial resistance.

## Figures and Tables

**Table 1:** Co-differentially expressed genes in *E. coli* ATCC25922 induced by 1/2× MIC and 1/10× MIC of OLA.

Gene Name	Function	Foldchange of Gene Expression	
		(CvB)	(DvB)
**Up-Regulated Genes**			
**1 Transcription Factors**			
*malT*	LuxR family transcriptional regulator, maltose regulon positive regulatory protein	6.29	8.50
*soxS*	AraC family transcriptional regulator, mar-sox-rob regulon activator	4.16	4.65
*mlc*	transcriptional regulator of PTS gene	4.02	3.15
**2 Oxidoreductases**			
*wrbA*	NAD(P)H dehydrogenase (quinone)	2.60	5.15
*ahpC*	alkyl hydroperoxide reductase	2.12	4.85
*cydB*	cytochrome bd ubiquinol oxidase subunit II	2.14	4.37
*DR76_76*	oxidation-reduction process; coenzyme binding; oxidoreductase activity; mannitol metabolic	2.60	3.68
*adhE*	acetaldehyde dehydrogenase/alcohol dehydrogenase	2.02	3.32
*icdA*	isocitrate dehydrogenase	2.80	3.09
*nuoM*	NADH-quinone oxidoreductase subunit M	2.30	3.07
*nuoI*	NADH-quinone oxidoreductase subunit N	2.34	2.95
*pfo*	pyruvate-ferredoxin/flavodoxin oxidoreductase	3.44	2.86
*grxB*	glutaredoxin 2	2.06	2.83
*fdoI*	formate dehydrogenase subunit gamma	2.24	2.29
*fdnG*	formate dehydrogenase major subunit	2.27	2.23
*gcvP*	glycine dehydrogenase	2.28	2.16
*fdoH*	formate dehydrogenase iron-sulfur subunit	2.14	2.11
**3 PTS system**			
*manX*	PTS system, mannose-specific IIAB component	9.39	15.64
*manZ*	PTS system, mannose-specific IID component	8.80	12.58
*ptsG*	glucose-specific PTS enzyme IIBC component	8.73	12.52
*manY*	PTS system, mannose-specific IIC component	8.60	10.25
*ptsI*	phosphotransferase system, enzyme I, PtsI	2.78	2.68
*ptsH*	phosphocarrier protein HPr	2.70	2.32
**4 Sugar and amino acid metabolism**			
*asnA*	aspartate-ammonia ligase	2.39	6.20
*asnB*	asparagine synthase (glutamine-hydrolysing)	2.27	4.30
*gcvT*	aminomethyl transferase	2.16	3.39
*aspC*	aspartate aminotransferase	2.01	3.27
*purA*	adenylosuccinate synthase	2.09	2.61
*fbaA*	fructose-bisphosphate aldolase, class II	2.49	2.31
**5 Others**			
*bssR*	biofilm regulator BssR	2.46	5.36
*feoB*	ferrous iron transport protein B	2.38	4.14
*iraD*	anti-adapter protein iraD	4.62	3.24
*pflD*	formate C-acetyltransferase	3.63	2.89
*zraS*	two-component system, NtrC family, sensor histidine kinase HydH	2.60	2.05
*DR76_3474*	uncharacterized protein	2.37	2.02
**Down-Regulated Genes**			
**1 DNA Binding and Repair**			
*pgaA*	DNA binding; negative regulation of DNA-templated transcription, termination	0	0
*rusA*	crossover junction endodeoxy ribonuclease RusA	0	0.0066
*hyaE*	DNA binding; DNA recombination	0	0
*putP*	DNA replication initiation	0.0015	0.0033
*hybD*	DNA recombination; DNA binding; DNA integration	0.0020	0.0037
*bet*	DNA binding; DNA metabolic process	0.0031	0.0017
*putA*	DNA replication	0.0036	0.00071
*exo*	DNA binding; nucleic acid phosphodiester bond hydrolysis; exonuclease activity	0.0038	0.0024
*umuC*	DNA polymerase V	0.35	0.39
**2 Membrane component**			
*ompD*	outer membrane porin protein LC	0.0013	0.0012
*DR76_3645*	integral component of membrane	0.0026	0
*DR76_3655*	integral component of membrane	0.0028	0.010
*cea*	cytolysis, integral component of membrane	0.28	0.47
**3 Others**			
*DR76_3678*	hypothetical protein	0	0.0051
*rutG*	pyrimidine utilization transport protein G	0.0047	0.0043
*yccM*	putative electron transport protein	0.014	0
*pepN*	aminopeptidase N	0.15	0.23
*higA*	toxin HigB-1	0.16	0.12
*motA*	chemotaxis protein MotA	0.39	0.48
*tsr*	methyl-accepting chemotaxis protein I, serine sensor receptor	0.48	0.48
*pspA*	phage shock protein A	0.48	0.23
*pspG*	phage shock protein G	0.36	0.18

Note: CvB means 1/2× MIC OLA treated group compared with untreated group; DvB means 1/10× MIC OLA treated group compared with untreated group.

**Table 2:** Genes with non-synonymous mutation and/or single-point deletion in *E. coli* ATCC25922 both induced by 1/2× MIC and 1/10× MICof OLA.

Gene Symbol	Gene Description	Mutations	Consequence
*degQ*	periplasmic serine protease, S1-C subfamily, contain C-terminal PDZ domain	1	NS
*ks71A*	hypothetical protein, regulation of transcription, DNA-templated	1	NS
*kduI*	4-deoxy-L-threo-5-hexosulose-uronate ketol-isomerase	1	SL
*DR76_2530*	type I phosphodiesterase / nucleotide pyrophosphatase family protein	4	SL
*gntP*	gluconate:H^+^ symporter, GntP family	1	NS
*cmoA*	iron complex transport system permease protein	1	NS
*vgrG*	type VI secretion system secreted protein VgrG	4	NS
*bigA*	putative surface-exposed virulence protein	1	NS
*DR76_3990*	hypothetical protein	1	NS
*pdxA*	4-hydroxythreonine-4-phosphate dehydrogenase PdxA	1	NS
*DR76_4321*	hypothetical protein	3	SL
*cusA*	Cu(I)/Ag(I) efflux system membrane protein CusA / SilA	1	NS
*DR76_4582*	hypothetical protein	1	NS
*DR76_4702*	integral component of membrane	1	NS
*queC*	threonine aldolase	1	NS

**Note:** NS, non-synonymous; SL, stop-loss, a mutation in the original termination codon (a stop was lost).

**Table 3:** Non-synonymous mutations in resistant *E. coli* ATCC25922 induced by sub-MIC of OLA.

Gene Name	Description	Mutations	Consequence
*intA*	prophage integrase IntA	1	NS
*murP*	PTS system, N-acetylmuramic acid-specific IIC component	1	NS
*nudK*	8-oxo-dGTP pyrophosphatase MutT and related house-cleaning NTP	2	NS
*yiaF*	uncharacterized protein YiaF	1	NS
*fimD*	outer membrane usher protein FimD/PapC	1	NS
*degQ*	periplasmic serine protease, S1-C subfamily, contain C-terminal PDZ domain	1	NS
*ks71A*	hypothetical protein; regulation of transcription, DNA-templated	1	NS
*yagK*	uncharacterized protein YagK	2	NS
*aas*	acyl-CoA synthetase (AMP-forming)/AMP-acid ligase II	1	NS
*srlA*	PTS system glucitol/sorbitol-specific EIIB component	1	NS
*ulaC*	di- and tricarboxylate transporter	1	NS
*pbμG*	xanthine/uracil/vitamin C permease, AzgA family	1	NS
*iraD*	DNA-binding transcriptional regulator, XRE-family HTH domain	1	NS
*yjhF*	H+/gluconate symporter or related permease	1	NS
*ydeE*	predicted transcriptional regulator YdeE, contains AraC-type DNA-binding domain	1	NS
*acnB*	aconitase B	1	NS
*htrE*	outer membrane usher protein FimD/PapC	1	NS
*rssA*	predicted acylesterase/phospholipase RssA, containd patatin domain	75	NS/SG
*crfC*	replication fork clamp-binding protein CrfC (dynamin-like GTPase family)	122	NS/SG/FSD/FSI/NFSI
*yeeP*	predicted GTPase	10	NS
*hmuU*	ABC-type Fe3+-siderophore transport system, permease component	1	NS
*yaiP*	glycosyltransferase, catalytic subunit of cellulose synthase and poly-beta-1,6-N-acetylglucosamine synthase	1	NS
*fliC*	flagellin and related hook-associated protein FlgL	1	NS
*yecM*	uncharacterized conserved protein YecM, predicted metalloenzyme	1	NS
*rnfD*	Na+-translocating ferredoxin:NAD+ oxidoreductase	1	NS
*rnfC*	Na+-translocating ferredoxin:NAD+ oxidoreductase RNF, RnfC subunit	14	NS/FSD/FSI
*mlc*	sugar kinase of the NBD/HSP70 family, may contain an N-terminal HTH domain	1	NS
*yddG*	ermease of the drug/metabolite transporter (DMT) superfamily	1	NS
*vgrG*	type VI secretion system secreted protein VgrG	2	NS
*bigA*	putative surface-exposed virulence protein	10	NS
*yciI*	uncharacterized conserved protein YciI, contains a putative active-site phosphohistidine	3	NS/FSD/FSI
*icd*	isocitrate dehydrogenase	2	NS
*DR76_4080*	minor capsid protein E	1	NS
*shp*	head decoration protein	2	NS
*sppA*	periplasmic serine protease, ClpP class	7	NS
*msyB*	acidic protein MsyB	1	NS
*yeeR*	inner membrane protein YeeR	2	NS
*yciC*	GTPase, G3E family	1	NS
*cdiA*	tRNA nuclease CdiA	7	NS/NFSD
*ltaE*	threonine aldolase	1	NS
*cusA*	Cu(I)/Ag(I) efflux system membrane protein CusA / SilA	1	NS
*acrB*	multidrug efflux pump subunit AcrB	1	NS
*yahD*	putative ankyrin repeat protein YahD	1	NS
*ycjY*	uncharacterized protein	1	NS
*DR76_4702*	integral component of membrane	1	NS
*fhaB*	large exoprotein involved in heme utilization or adhesion	4	NS
*yjcZ*	uncharacterized protein YjcZ	3	NS
*stfR*	phage tail fiber repeat family protein	1	NS
*lrhA*	probable HTH-type transcriptional regulator LrhA	1	FSD
*rsxC*	electron transport complex subunit RsxC	1	NFSD

**Notes:** NS, non-synonymous; SG, stop gain; FSD, frameshift deletion; NFSD, non-frameshift deletion; FSI, frameshift insertion; NFSI, non-frameshift insertion.

**Table 4:** The MICs of antimicrobial agents against the wildtype and OLA resistant *E. coli* ATCC25922. (μg/mL)

Antibiotics	Wildtype	8× MIC OLA ^R^ (1/2)	8× MIC OLA ^R^ (1/10)
Ampicillin	4	16	16
Augmentin (Amoxicillin+Clavulanic acid)	4/2	8/4	8/4
Gentamicin	1	0.5	1
Tetracycline	1	2	2
Spectinomycin	32	16	16
Florfenicol	4	16	16
Sulfaisoxazole	16	32	32
Compound sulfamethoxazole (Sulfamethoxazole +Trimethoprim)	0.06/1.2	0.25/4.8	0.25/4.8
Ceftiofur	0.5	0.5	0.5
Ceftazidime	1	2	2
Enrofloxacin	0.015	0.03	0.03
Ofloxacin	0.06	0.125	0.125
Meropenem	0.03	0.03	0.03
Apramycin	32	32	32
Colistin	2	0.5	0.5
Mequindox	8	64	64

## Supplementary Materials

The following are available online at https://www.mdpi.com/2079-6382/9/11/791/s1, Figure S1: Resistance rates of *E. coli* ATCC25922 exposed to ENR at sub-MIC concentrations of 1/2× MIC (A), 1/4× MIC (B), 1/10× MIC (C) and 1/100× MIC (D), Figure S2. Scanning electron micrograph of *E. coli* ATCC25922 induced by none (A), 1/2× MIC ENR (B) and 1/2× MIC OLA(C)., Figure S3. Correlation tests for 9 samples (triplicates in each group) of the untreated (group B), 1/2× MIC (group C) and 1/10× MIC OLA (group D) -treated *E. coli* ATCC25922. The abscissa and ordinate in the figure are sample numbers. The closer the block value is to 1, the higher the similarity is., Figure S4. Venn diagrams of DEGs. The numbers in each circle represents the total number of DEGs in the comparison combination, and the overlapping part of the circles represents the DEGs shared between the comparison groups. B, untreated group; C, 1/2× MIC OLA-treated group; D, 1/10× MIC OLA-treated group., Figure S5. The genome of 1/2× MIC OLA induced *E. coli* ATCC25922 resistant to 8× MIC OLA. The circles from outermost to innermost indicated the scale, GC content (red: > average value, blue: < average value), GC skew (purple: >0, orange: <0), noncoding RNA (tRNA in black, rRNA in red), minus-stranded CDS maps, plus-stranded CDS maps, minus-stranded base-modified map (full red circle), plus-stranded base-modified map (full blue circle), gene map of the restriction modification system, respectively., Figure S6. Protein length distribution of the genome of OLA resistant *E. coli* ATCC25922., Figure S7. COG function classification of OLA resistant *E. coli* ATCC25922. The abscissa is the group of the COG, and the ordinate is the number of genes annotated to the group., Figure S8. GO function annotation of OLA resistant *E. coli* ATCC25922. The ordinate is the three major classes of the GO term including biological processes, cellular components, and molecular functions, the abscissa is the number of genes annotated to the term (including the term of the term)., Figure S9. KEGG functions annotation of OLA resistant *E. coli* ATCC25922. The ordinate is the name of the KEGG metabolic pathway, and the abscissa is the number of genes annotated to the pathway. The genes are divided into five branches according to the involved KEGG metabolic pathway including Cellular Processes, Environmental Information Processing, Genetic Information Processing, Metabolism, and Organic Systems., Table S1: Table S1. Data Filtering Statistics, Table S2. RNASeq Map Statistics., Table S3. Reference genome annotation information statistics., Table S4. Number of differentially expressed genes between groups B and C., Table S5. Differentially expressed genes between groups B and D.

## Abbreviations

CFU	Colony-Forming Units
DEGs	Differentially expressed genes
*E. coli*	*Escherichia coli*
ENR	Enrofloxacin
MIC	Minimum inhibitory concentration
NADH	Nicotinamide adenine dinucleotide
OLA	Olaquindox
PEP	phosphoenolpyruvate
PTS	Phosphoenolpyruvate-sugar phosphotransferase system
QdNOs	Quinoxaline1,4-di-*N*-oxides
SNP	Single Nucleotide Polymorphisms
SSH PCR	Suppression subtractive hybridization polymerase chain reaction
Sub-MIC	Sub-inhibitory concentration
TCA	tricarboxylic acid cycle

## References

[1] Woolhouse M.E.J., Waugh C., Perry M.R., Nair H. (2016). Global disease burden due to antibiotic resistance—State of the evidence. J. Glob. Health.

[2] Julian D., Spiegelman G.B., Grace Y. (2006). The world of subinhibitory antibiotic concentrations. Curr. Opin. Microbiol..

[3] D’Costa V.M., King C.E., Lindsay K., Mariya M., Sung W.W.L., Carsten S., Duane F., Grant Z., Fabrice C., Regis D. (2011). Antibiotic resistance is ancient. Nature.

[4] Dan I.A., Diarmaid H. (2014). Microbiological effects of sublethal levels of antibiotics. Nat. Rev. Microbiol..

[5] Hao H.H., Cheng G.Y., Iqbal Z., Ai X.H., Hussain H.I., Huang L.L., Dai M.H., Wang Y.L., Liu Z.L., Yuan Z.H. (2014). Benefits and risks of antimicrobial use in food-producing animals. Front. Microbiol..

[6] Zeynep B., Didier M. (2015). SOS, the formidable strategy of bacteria against aggressions. FEMS Microbiol. Rev..

[7] Dan I.A., Hughes D. (2012). Evolution of antibiotic resistance at non-lethal drug concentrations. Drug Resist. Updat..

[8] Diarmaid H., Dan I., Andersson J. (2012). Selection of resistance at lethal and non-lethal antibiotic concentrations. Curr. Opin. Microbiol..

[9] Laureti L., Matic I., Gutierrez A.J.A. (2013). Bacterial Responses and Genome Instability Induced by Subinhibitory Concentrations of Antibiotics. Antibiotics.

[10] Erik G., Sha C., Berg O.G., Carolina I.C., Linus S., Diarmaid H., Dan I.A. (2011). Selection of resistant bacteria at very low antibiotic concentrations. PLoS Pathog..

[11] Cairns J., Becks L., Jalasvuori M., Hiltunen T. (2016). Sublethal streptomycin concentrations and lytic bacteriophage together promote resistance evolution. Philos. Trans. R Soc. Lond. B Biol. Sci..

[12] Carta A., Corona P., Loriga M. (2005). Quinoxaline 1,4-Dioxide: A Versatile Scaffold Endowed With Manifold Activities. Curr. Med. Chem..

[13] Guo W., Hao H., Dai M., Wang Y., Huang L., Peng D., Wang X., Wang H., Yao M., Sun Y.J.P.O. (2012). Development of quinoxaline 1, 4-dioxides resistance in Escherichia coli and molecular change under resistance selection. PLoS ONE.

[14] Duan Z., Yi J., Fang G., Fan L., Wang S.J.F.C. (2013). A sensitive and selective imprinted solid phase extraction coupled to HPLC for simultaneous detection of trace quinoxaline-2-carboxylic acid and methyl-3-quinoxaline-2-carboxylic acid in animal muscles. Food Chem..

[15] (2008). Prevalence and patterns of antimicrobial resistance of fecal Escherichia coli among pigs on 47 farrow-to-finish farms with different in-feed medication policies in Ontario and British Columbia. Can. J. Vet. Res. Rev. Can. Rech. Vet..

[16] Sørensen A.H., Hansen L.H., Johannesen E., Sørensen S.J. (2003). Conjugative plasmid conferring resistance to olaquindox. Antimicrob. Agents Chemother..

[17] Hansen L.H., Johannesen E., Burmolle M., Sorensen A.H., Sorensen S.J. (2004). Plasmid-Encoded Multidrug Efflux Pump Conferring Resistance to Olaquindox in Escherichia coli. Antimicrob. Agents chemother..

[18] Hansen L.H., Jensen L.B., Sørensen H.I., Sørensen S.J. (2007). Substrate specificity of the OqxAB multidrug resistance pump in Escherichia coli and selected enteric bacteria. J. Antimicrob. Chemother..

[19] Haihong H., Weige G., Zahid I., Guyue C., Xu W., Menghong D., Lingli H., Yulian W., Dapeng P., Zhenli L. (2013). Impact of cyadox on human colonic microflora in chemostat models. Regul. Toxicol. Pharmacol..

[20] Suter W., Rosselet A., Knüsel F. (1978). Chemotherapy. Mode of action of quindoxin and substituted quinoxaline-di-N-oxides on Escherichia coli. J. Antimicrob. Agents.

[21] Lindgren P.K., Karlsson A., Hughes D. (2003). Mutation Rate and Evolution of Fluoroquinolone Resistance in Escherichia coli Isolates from Patients with Urinary Tract Infections. Antimicrob. Agents Chemother..

[22] Kohanski M.A., Depristo M.A., Collins J.J. (2010). Sublethal Antibiotic Treatment Leads to Multidrug Resistance via Radical-Induced MutagPenesis. Mol Cell.

[23] Ren D., Bedzyk L.A., Thomas S.M., Ye R.W., Wood T.K. (2004). Biotechnology. Gene expression in Escherichia coli biofilms. J. Appl. Microbiol..

[24] Joanna D., Jintae L., Wood T.K.E. (2006). YliH (BssR) and YceP (BssS) regulate Escherichia coli K-12 biofilm formation by influencing cell signaling. J. Appl. Microbiol..

[25] Szybalski W., Bryson V. (1952). Genetic studies on microbial cross resistance to toxic agents. I. Cross resistance of Escherichia coli to fifteen antibiotics. J. Bacteriol..

[26] Nicole L.P., Elizabeth G.A.F., Julia K., Sørum V., Primicerio R., Roberts A.P., Rozen D.E., Samuelsen Ø., Johnsen P.J. (2018). Conserved collateral antibiotic susceptibility networks in diverse clinical strains of Escherichia coli. Nat. Commun..

[27] Pál C., Papp B., Lázár V. (2015). Collateral sensitivity of antibiotic-resistant microbes. Trends Microbiol..

[28] Plumbridge J. (2002). Regulation of gene expression in the PTS in Escherichia coli: The role and interactions of Mlc. Curr. Opin. Microbiol..

[29] Larquet E., Schreiber V., Boisset N., Richet E.J.J.O.M.B. (2004). Oligomeric Assemblies of the Escherichia coli MalT Transcriptional Activator Revealed by Cryo-electron Microscopy and Image Processing. J. Mol. Biol..

[30] Eisenreich W., Dandekar T., Heesemann J., Goebel W.J.N.R.M. (2010). Carbon metabolism of intracellular bacterial pathogens and possible links to virulence. Nat. Rev. Microbiol..

[31] Leonardo M.R., Dailly Y., Clark D.P. (1996). Role of NAD in Regulating the adhE Gene of Escherichia coli. J. Bacteriol..

[32] Echave Lozano P., Tamarit Sumalla J., Cabiscol Català E., Ros Salvador J. (2003). Novel Antioxidant Role of Alcohol Dehydrogenase E from Escherichia coli. J. Biol. Chem..

[33] McNeely K., Yu X., Ananyev G., Bennette N., Bryant D.A., Dismukes G.C. (2011). Synechococcus sp. strain PCC 7002 nifJ mutant lacking pyruvate: Ferredoxin oxidoreductase. Appl. Environ. Microbiol..

[34] Patridge E.V., Ferry J.G. (2006). WrbA from Escherichia coli and Archaeoglobus fulgidus is an NAD(P)H:quinone oxidoreductase. J. Bacteriol..

[35] Wolfova J., Smatanova I.J., Mesters J.R., Lapkouski M., Kuty M., Natalello A., Chatterjee N., Chern S.Y., Ebbel E., Ricci A. (2009). Structural organization of WrbA in apo- and holoprotein crystals. Biochim. Et Biophys. Acta Proteins Proteom..

[36] Mark S., Guido S., Cook G.M., Poole R.K. (2010). Compensations for diminished terminal oxidase activity in Escherichia coli: Cytochrome bd-II-mediated respiration and glutamate metabolism. J. Biol. Chem..

[37] Alessandro G., Borisov V.B., Marzia A., Paolo S., Elena F. (2014). Cytochrome bd oxidase and bacterial tolerance to oxidative and nitrosative stress. Biochim. Biophys. Acta.

[38] Friedrich T., Dekovic D.K., Burschel S. (2016). Assembly of the Escherichia coli NADH: Ubiquinone oxidoreductase (respiratory complex I). Bba Bioenerg..

[39] Chen C., Cheng G., Hao H., Dai M., Wang X., Huang L., Liu Z., Yuan Z. (2013). Mechanism of Porcine Liver Xanthine Oxidoreductase Mediated N-Oxide Reduction of Cyadox as Revealed by Docking and Mutagenesis Studies. PloS ONE.

[40] Semchyshyn H., Bagnyukova T., Lushchak V. (2005). Involvement of soxRS regulon in response of Escherichia coli to oxidative stress induced by hydrogen peroxide. Biochem. Biokhimiia.

[41] Chubiz A., Glekas G.D., Rao C.V. (2012). Transcriptional Cross Talk within the mar-sox-rob Regulon in Escherichia coli Is Limited to the rob and rnarRAB Operons. J. Bacteriol..

[42] Valérie Duval I.M.L. (2013). MarA, SoxS and Rob of Escherichia coli*—*Global regulators of multidrug resistance, virulence and stress response. Int. J. Biotechnol. Wellness Ind..

[43] Siedler S., Schendzielorz G., Binder S., Eggeling L., Bringer S., Bott M. (2014). SoxR as a Single-Cell Biosensor for NADPH-Consuming Enzymes in Escherichia coli. ACS Synthetic Biol..

[44] Michael R., Jacques M., Poole L.B. (2002). An NADH-dependent bacterial thioredoxin reductase-like protein in conjunction with a glutaredoxin homologue form a unique peroxiredoxin (AhpC) reducing system in Clostridium pasteurianum. Biochemistry.

[45] Guimar B.G., Hélène S., Nadine H., Brigitte S.J., Roland B., William S., Cole S.T., Alzari P.M. (2005). Structure and mechanism of the alkyl hydroperoxidase AhpC, a key element of the Mycobacterium tuberculosis defense system against oxidative stress. J. Biol. Chem..

[46] Alexios V.G., Aristi P., Raz Z., Ayala H., Arne H. (2002). Characterization of Escherichia coli null mutants for glutaredoxin 2. J. Biol. Chem..

[47] Iwadate Y., Funabasama N., Kato J.I. (2017). Involvement of formate dehydrogenases in stationary phase oxidative stress tolerance in Escherichia coli. FEMS Microbiol. Lett..

[48] Thomas S.C., Alhasawi A., Auger C., Omri A., Appanna V.D. (2016). The role of formate in combatting oxidative stress. Antonie Van Leeuwenhoek.

[49] Cheng G., Li B., Wang C., Zhang H., Liang G., Weng Z., Wang X., Liu Z., Dai M. (2015). Systematic and Molecular Basis of the Antibacterial Action of Quinoxaline 1,4-Di-N-Oxides against Escherichia coli. PloS ONE.

[50] Kuban W., Vaisman A., Mcdonald J.P., Karata K., Yang W., Goodman M.F., Woodgate R. (2012). Escherichia coli UmuC active site mutants: Effects on translesion DNA synthesis, mutagenesis and cell survival. DNA Repair.

[51] Lilley D.M., White M.F. (2000). Resolving the relationships of resolving enzymes. J. Proc. Natl. Acad. Sci. USA.

[52] Botou M., Lazou P., Papakostas K., Lambrinidis G., Evangelidis T., Mikros E., Frillingos S. (2018). Insight on specificity of uracil permeases of the NAT/NCS2 family from analysis of the transporter encoded in the pyrimidine utilization operon of Escherichia coli. Mol. Microbiol..

[53] Nadine H.N., J Merijn S., Stanley B., Kuile B.H. (2013). Chemotherapy. Compensation of the metabolic costs of antibiotic resistance by physiological adaptation in Escherichia coli. J. Antimicrob. Agents.

[54] Zheng M., Wang X., Templeton L.J., Smulski D.R., Larossa R.A., Storz G. (2001). DNA microarray-mediated transcriptional profiling of the Escherichia coli response to hydrogen peroxide. J. Bacteriol..

[55] Aoki S.K., Webb J.S., Braaten B.A., Low D.A. (2009). Contact-dependent growth inhibition causes reversible metabolic downregulation in Escherichia coli. J. Bacteriol..

[56] Wang J., Zhou Z., He F., Ruan Z., Jiang Y., Hua X., Yu Y. (2018). The role of the type VI secretion system vgrG gene in the virulence and antimicrobial resistance of Acinetobacter baumannii ATCC 19606. PLoS ONE.

[57] Mikiro H., Kazuhiko T., Makoto Y., Yoshiyuki Y. (2010). Effect of multidrug-efflux transporter genes on dipeptide resistance and overproduction in Escherichia coli. FEMS Microbiol. Lett..

[58] Foti J.J., Babho D., Winkler J.A., Collins J.J., Walker G.C. (2012). Oxidation of the guanine nucleotide pool underlies cell death by bactericidal antibiotics. Science.

[59] Ozaki S., Matsuda Y., Keyamura K., Kawakami H., Noguchi Y., Kasho K., Nagata K., Masuda T., Sakiyama Y., Katayama T. (2013). A replicase clamp-binding dynamin-like protein promotes colocalization of nascent DNA strands and equipartitioning of chromosomes in E. coli. Cell Rep..

[60] Volokhina E.B., Jan G., Michiel S., Ingrid S., Jan T., Bos M.P. (2011). Role of the periplasmic chaperones Skp, SurA, and DegQ in outer membrane protein biogenesis in Neisseria meningitidis. J. Bacteriol..

[61] Justyna S., Hélène M., Tobias K., Flavia C., Michael E., Tim C. (2011). Molecular adaptation of the DegQ protease to exert protein quality control in the bacterial cell envelope. J. Biol. Chem..

[62] Rhen M., Van Die I., Rhen V., Bergmans H. (2010). Comparison of the nucleotide sequences of the genes encoding the KS71A and F7(1) fimbrial antigens of uropathogenic Escherichia coli. Eur. J. Biochem.

[63] Czibener C., Merwaiss F., Guaimas F., Del Giudice M.G., Serantes D.A., Spera J.M., Ugalde J.E. (2016). BigA is a novel adhesin of Brucella that mediates adhesion to epithelial cells. Cell Microbiol..

[64] Meir A., Abdelhai A., Moskovitz Y., Ruthstein S. (2017). EPR Spectroscopy Targets Structural Changes in the E.coli Membrane Fusion CusB upon Cu(I) Binding. Biophys. J..

[65] Kohanski M.A., Dwyer D.J., Hayete B., Lawrence C.A., Collins J.J. (2007). A Common Mechanism of Cellular Death Induced by Bactericidal Antibiotics. Cell.

[66] Clinical and Laboratory Standards Institute (2009). Method for Dilution Antimicrobial Susceptibility Test for bacteria that grow aerobically. Approved Standard.

[67] Xu F., Cheng G., Hao H., Wang Y., Wang X., Chen D., Peng D., Liu Z., Yuan Z., Dai M. (2016). Mechanisms of Antibacterial Action of Quinoxaline 1,4-di-N-oxides against Clostridium perfringens and Brachyspira hyodysenteriae. Front. Microbiol..

[68] Amit K.T., Danka B., Davide G., Milan V., Maria Elisabetta G., Anushree M., Marin P.D. (2013). Antimicrobial Potential and Chemical Characterization of Serbian Liverwort (Porella arboris-vitae): SEM and TEM Observations. Evid. Based Complement. Altern. Med..

[69] Moskowitz S.M., Foster J.M., Julia E., Burns J.L. (2004). Clinically feasible biofilm susceptibility assay for isolates of Pseudomonas aeruginosa from patients with cystic fibrosis. J. Clin. Microbiol..

[70] Grabherr M.G., Haas B.J., Moran Y., Levin J.Z., Thompson D.A., Ido A., Xian A., Lin F., Raktima R., Qiandong Z. (2011). Full-length transcriptome assembly from RNA-Seq data without a reference genome. Nat. Biotechnol..

[71] Jombart T. (2008). adegenet: A R package for the multivariate analysis of genetic markers. Bioinformatics.

[72] Aparicio G., Götz S., Conesa A., Segrelles D., Blanquer I., García J.M., Hernandez V., Robles M., Talon M. (2006). Blast2GO goes grid: Developing a grid-enabled prototype for functional genomics analysis. Stud. Health Technol. Inform..

[73] Koren S., Walenz B.P., Berlin K., Miller J.R., Bergman N.H., Phillippy A.M. (2017). Canu: Scalable and accurate long-read assembly via adaptive k-mer weighting and repeat separation. Genome Res..

[74] Delcher A.L., Harmon D., Kasif S., White O., Salzberg S.L. (1999). Improved microbial gene identification with GLIMMER. Nucleic Acids Res..

[75] Lowe T.M., Eddy S.R. (1997). tRNAscan-SE: A program for improved detection of transfer RNA genes in genomic sequence. Nucleic Acids Res..

[76] Lagesen K., Hallin P., Rodland E., Staerfeldt H., Rognes T., Ussery D. (2007). RNAmmer: Consistent and rapid annotation of ribosomal RNA genes. Nucleic Acids Res..

[77] Sam G.J., Simon M., Mhairi M., Ajay K., Eddy S.R., Alex B. (2005). Rfam: Annotating non-coding RNAs in complete genomes. Nucleic Acids Res..

[78] Benson G. (1999). Tandem repeats finder: A program to analyze DNA sequences. Nucleic Acids Res..

[79] Bland C., Ramsey T.L., Sabree F., Lowe M., Brown K., Kyrpides N.C., Hugenholtz P. (2007). CRISPR Recognition Tool (CRT): A tool for automatic detection of clustered regularly interspaced palindromic repeats. BMC Bioinform..

[80] Cheng G., Sa W., Cao C., Guo L., Hao H., Liu Z., Wang X., Yuan Z. (2016). Quinoxaline 1,4-di-N-Oxides: Biological Activities and Mechanisms of Actions. Front. Pharmacol..

