# Supplementary material for: Development of Resistance in Escherichia coli ATCC25922 under Exposure of Sub-Inhibitory Concentration of Olaquindox
Source: Antibiotics (Basel). 2020 Nov 10;9(11):791. doi: 10.3390/antibiotics9110791 (PMC7696260; doi:10.3390/antibiotics9110791)
Supplement: Supplementary file 1 [file antibiotics-09-00791-s001.pdf]

Supplementary Materials:

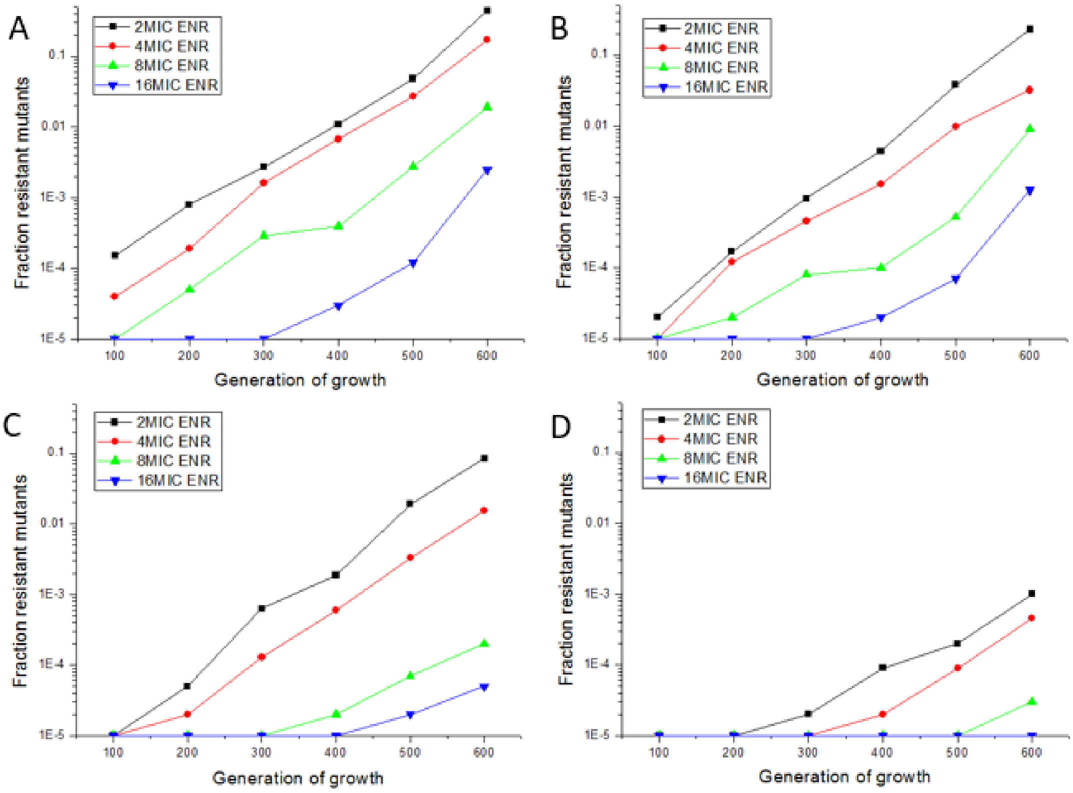

Figure S1. Resistance rates of *E. coli* ATCC25922 exposed to ENR at sub-MIC concentrations of 1/2×MIC (A), 1/4×MIC (B), 1/10×MIC (C) and 1/100×MIC (D).

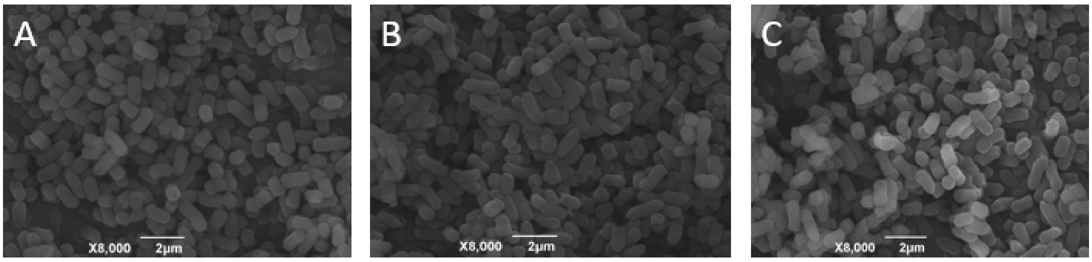

Figure S2. Scanning electron micrograph of *E. coli* ATCC25922 induced by none (A), 1/2×MIC ENR (B) and 1/2×MIC OLA(C).

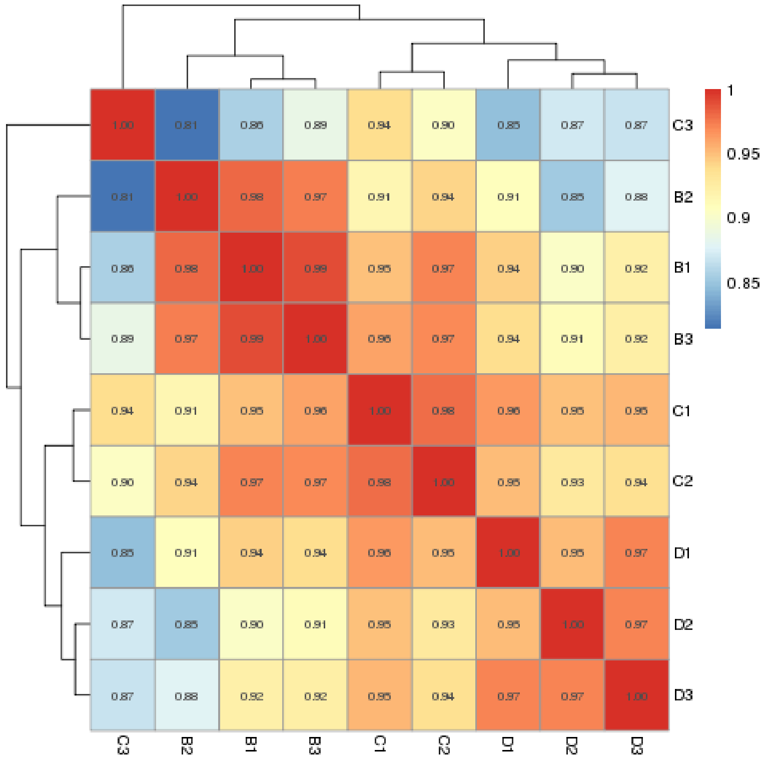

Figure S3. Correlation tests for 9 samples (triplicates in each group) of the untreated (group B), 1/2×MIC (group C) and 1/10×MIC OLA (group D) -treated *E. coli* ATCC25922. The abscissa and ordinate in the figure are sample numbers. The closer the block value is to 1, the higher the similarity is.

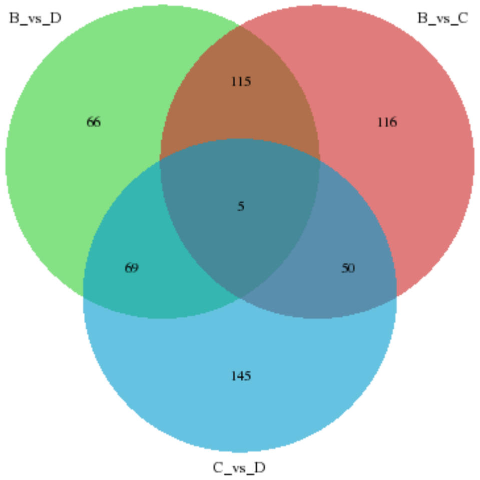

Figure S4. Venn diagrams of DEGs. The numbers in each circle represents the total number of DEGs in the comparison combination, and the overlapping part of the circles represents the DEGs shared between the comparison groups. B, untreated group; C, 1/2×MIC OLA-treated group; D, 1/10×MIC OLA-treated group.

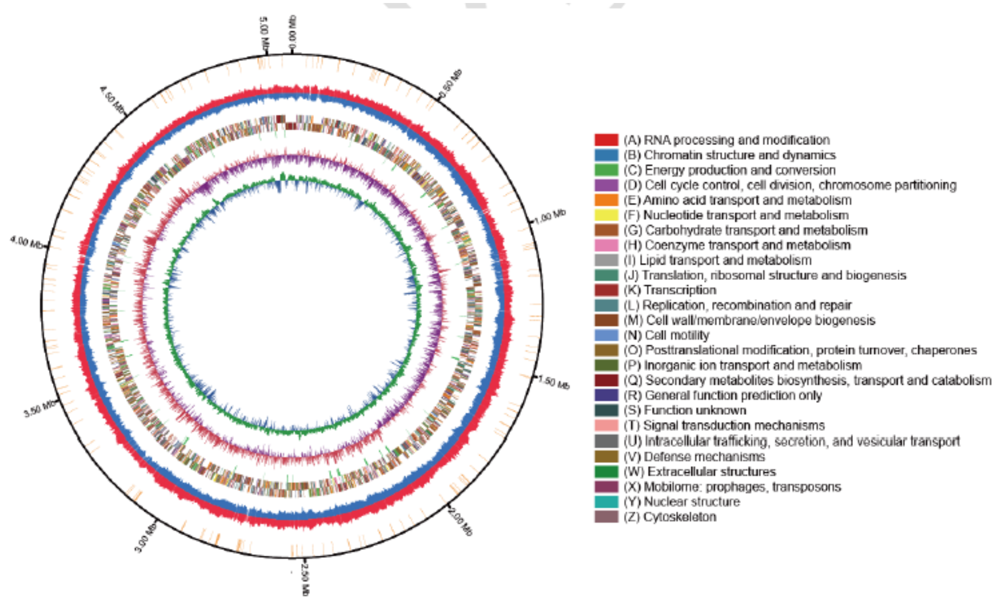

Figure S5. The genome of 1/2×MIC OLA induced *E. coli* ATCC25922 resistant to 8×MIC OLA. The circles from outermost to innermost indicated the scale, GC content (red: > average value, blue: < average value), GC skew (purple: >0, orange: <0), noncoding RNA (tRNA in black, rRNA in red), minus-stranded CDS maps, plus-stranded CDS maps, minus-stranded base-modified map (full red circle), plus-stranded base-modified map (full blue circle), gene map of the restriction modification system, respectively.

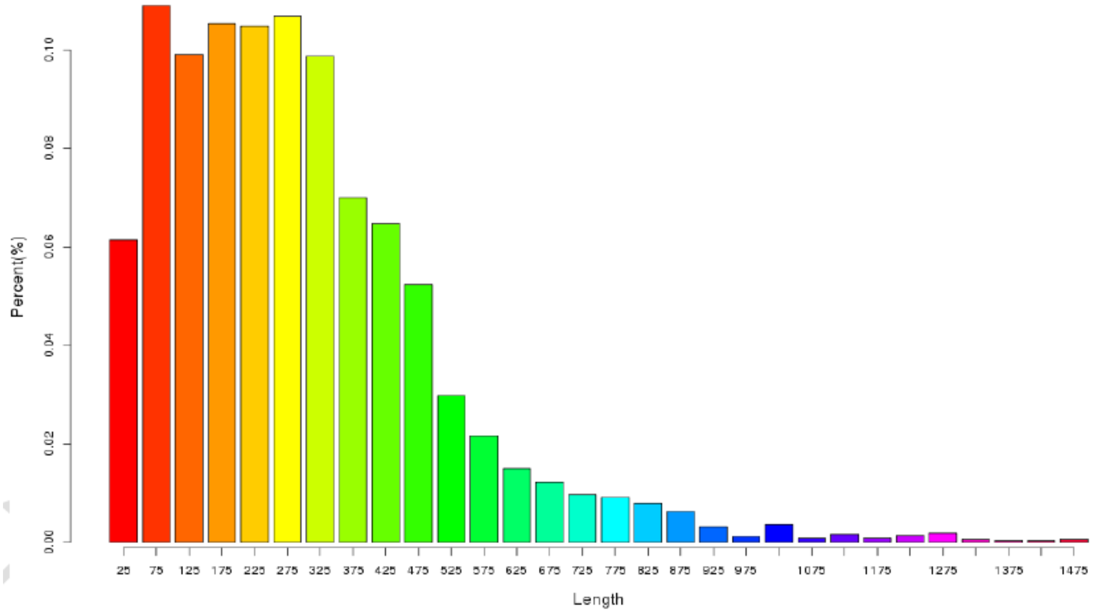

Figure S6. Protein length distribution of the genome of OLA resistant *E. coli* ATCC25922

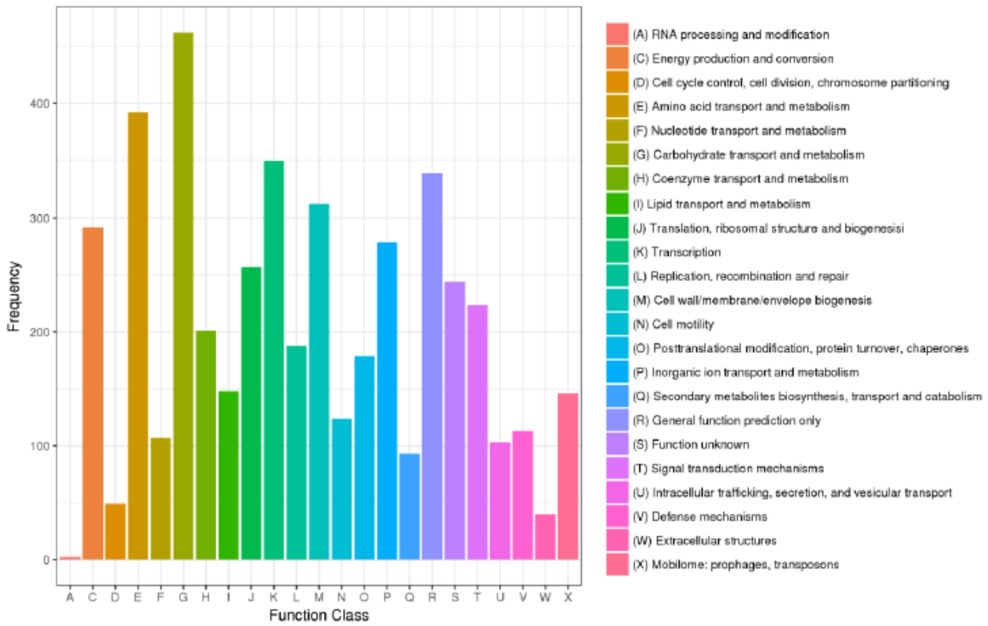

Figure S7. COG function classification of OLA resistant *E. coli* ATCC25922. The abscissa is the group of the COG, and the ordinate is the number of genes annotated to the group.

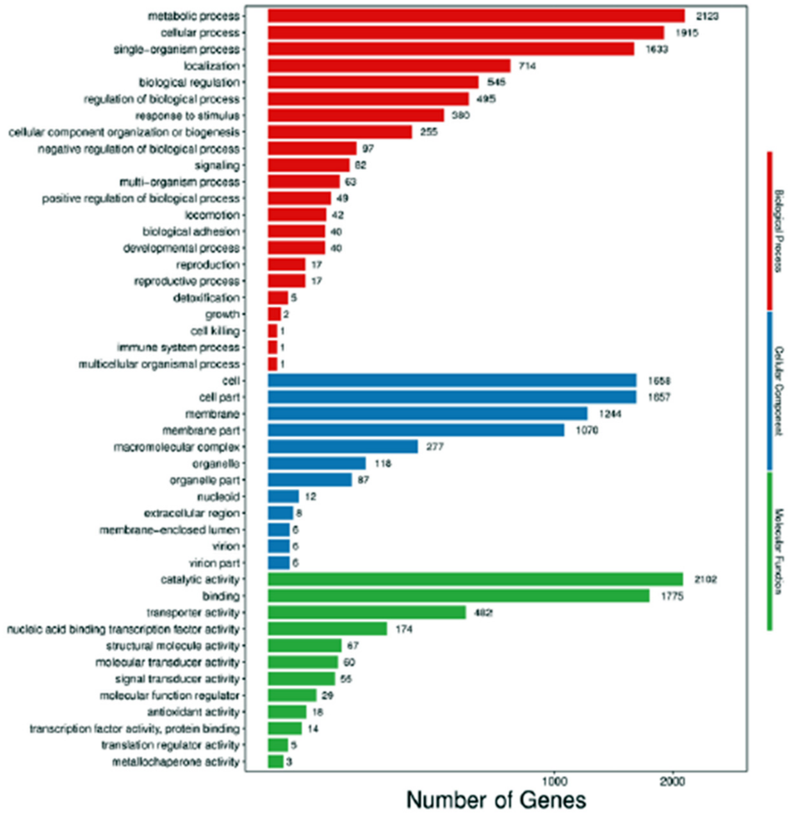

Figure S8. GO function annotation of OLA resistant *E. coli* ATCC25922. The ordinate is the three major classes of the GO term including biological processes, cellular components, and molecular functions, the abscissa is the number of genes annotated to the term (including the term of the term).

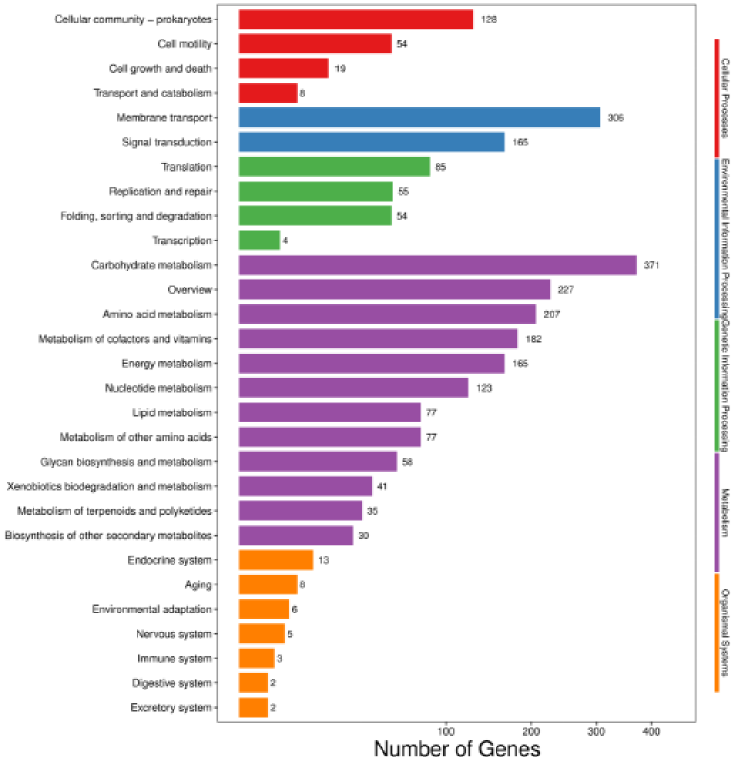

Figure S9. KEGG functions annotation of OLA resistant *E. coli* ATCC25922. The ordinate is the name of the KEGG metabolic pathway, and the abscissa is the number of genes annotated to the pathway. The genes are divided into five branches according to the involved KEGG metabolic pathway including Cellular Processes, Environmental Information Processing, Genetic Information Processing, Metabolism, and Organic Systems.

Table S1. Data Filtering Statistics

Sample	Clean Reads No.	Clean Data (bp)	GC (%)	Clean Reads (%)	Clean Data (%)
B1	21,505,222	3,132,423,838	52.92	97.69	94.23
B2	22,975,598	3,332,505,598	53.03	98.05	94.18
B3	18,093,076	2,530,130,532	52.42	96.83	89.67
C1	21,409,762	3,076,509,184	53.06	97.41	92.70
C2	22,948,696	3,324,945,742	52.98	97.47	93.52
C3	22,830,266	3,245,333,274	53.35	97.93	92.19
D1	21,241,676	3,075,246,532	53.35	98.25	94.20
D2	22,057,924	3,184,275,786	52.24	97.84	93.53
D3	18,162,483	2,657,601,052	52.94	95.92	92.95

Notes: Sample, sample name; Clean Reads No., high quality sequence; Clean Data (bp), high quality sequence base number; Clean Reads (%), high quality sequence reads as a percentage of sequencing reads; Clean Data (%), high-quality sequence bases as a percentage of sequencing bases.

Table S2. RNASeq Map Statistics

Sample	Useful Reads	Total Mapped Reads	(%)	Uniquely Mapped Reads	(%)	Multiple Mapped Reads	(%)
B1	21,505,222	20,978,534	97.55	20,320,467	96.86	658,067	3.14
B2	22,975,598	22,435,364	97.65	21,826,743	97.29	608,621	2.71
B3	18,093,076	17,684,324	97.74	17,009,674	96.19	674,650	3.81
C1	21,409,762	20,506,503	95.78	19,990,794	97.49	515,709	2.51

C2	22,948,696	22,553,947	98.28	21,954,418	97.34	599,529	2.66
C3	22,830,266	22,452,343	98.34	20,715,393	92.26	1,736,950	7.74
D1	21,241,676	20,786,599	97.86	20,153,578	96.95	633,021	3.05
D2	22,057,924	21,577,714	97.82	21,022,718	97.43	554,996	2.57
D3	18,162,438	17,678,749	97.34	17,319,672	97.97	359,077	2.03

Notes: Sample, sample name; Useful Reads, the total number of sequences used for comparison; Total Mapped Reads, the total number mapped on the reference genome; Uniquely Mapped Reads, the total number of uniquely mapped on the reference genome; Multiple Mapped Reads, , the total number of multiple mapped on the reference genome.

Table S3. Reference genome annotation information statistics

Database	Number	Percentage (%)
eggNOG Class	659	13.09
KO	3,183	63.22
Swissprot	4,367	86.73
NR	5,023	99.76
GO	2,489	49.43
All	5,035	100

Table S4. Number of differentially expressed genes between groups B and C

Gene ID	Gene name	Foldchange(CvB)	Pval	Regulation
gene1037	hdeB	0.416173604	0.039349249	Down Regulation
gene1149	malT	6.286487949	6.57171E-19	Up Regulation
gene1159	feoB	2.376601676	0.027054731	Up Regulation
gene1547	tolC	2.111180071	0.00084329	Up Regulation
gene1820	epd	2.003655097	0.000521886	Up Regulation
gene1822	fbaA	2.485279871	4.6938E-06	Up Regulation
gene1842	gcvT	2.163249735	0.032429735	Up Regulation
gene1844	gcvP	2.27532496	0.027932863	Up Regulation
gene1892	lysA	3.850194609	0.024807682	Up Regulation
gene2078	DR76_1951	2.338008596	0.003046503	Up Regulation
gene2079	ygaZ	2.170577798	0.004621548	Up Regulation
gene208	nuoN	2.158540486	0.000223431	Up Regulation
gene209	nuoM	2.295157503	7.15188E-05	Up Regulation
gene210	nuoL	2.569717925	3.43364E-06	Up Regulation
gene213	nuoI	2.339008465	0.000110098	Up Regulation
gene214	nuoH	2.729023109	2.11138E-06	Up Regulation
gene215	nuoG	2.26741505	4.11791E-05	Up Regulation
gene2160	thiS	3.14138092	0.017217506	Up Regulation
gene2161	thiF|	2.262346122	0.000119187	Up Regulation
gene2162	thiE	2.139986273	0.000230549	Up Regulation
gene2163	thiC	2.213468175	4.92705E-05	Up Regulation
gene2173	zraS	2.604350749	1.52459E-06	Up Regulation
gene2188	iclR	2.080879956	0.000192758	Up Regulation
gene2231	pspG	0.359895166	0.000328609	Down Regulation
gene2262	soxS	4.158545845	1.52674E-12	Up Regulation
gene2315	phnG	0.219241071	0.005398822	Down Regulation
gene2328	basR	2.671254039	0.039716491	Up Regulation
gene2391	hfq	2.059921976	0.000229179	Up Regulation
gene2397	nsrR	2.046323258	0.00041658	Up Regulation
gene244	purF	2.157215022	0.004765102	Up Regulation
gene2475	argF	8.005906251	3.55483E-05	Up Regulation

gene2476	arcCǀ	4.750575728	0.018801951	Up Regulation
gene2477	arcA	2.914313089	0.032660989	Up Regulation
gene2637	tsr	0.478635922	0.000235577	Down Regulation
gene2689	thrA	3.342049743	1.80672E-09	Up Regulation
gene2690	thrB	2.253549625	4.60453E-05	Up Regulation
gene3085	msrQ	0.499051058	0.018474372	Down Regulation
gene3172	motA	0.393433092	3.58693E-05	Down Regulation
gene3173	motB	0.404071743	0.002500896	Down Regulation
gene3250	manZ	8.800461645	1.65998E-24	Up Regulation
gene3251	manY	8.596871218	6.51662E-24	Up Regulation
gene3252	manX	9.392500062	6.22114E-26	Up Regulation
gene3290	gap	2.700035318	0.000434982	Up Regulation
gene3356	thrS	2.007502552	0.00032741	Up Regulation
gene339	ptsH	2.7027774	7.2138E-07	Up Regulation
gene3391	DR76_3151	2.673977789	0.007110679	Up Regulation
gene340	ptsI	2.782279985	2.62405E-07	Up Regulation
gene3452	rnfD	0.476105879	0.007072053	Down Regulation
gene3493	mlc	4.024907347	6.21121E-07	Up Regulation
gene3535	cspG	2.394808573	0.040079345	Up Regulation
gene3587	marR	0.478579648	0.033977691	Down Regulation
gene3630	maeA	2.003989832	0.000349032	Up Regulation
gene3632	DR76_3370	0.151472946	0.000851592	Down Regulation
gene3633	higA	0.162976176	0.00082569	Down Regulation
gene3718	nifJ	3.438333246	1.00445E-09	Up Regulation
gene3769	pspA	0.483089373	0.008597387	Down Regulation
gene3801	cysBǀ	2.375078789	1.14824E-05	Up Regulation
gene3815	trpD	2.846458913	1.41921E-07	Up Regulation
gene3816	trpC	2.830590427	1.74902E-07	Up Regulation
gene3838	adhE	2.019595092	0.011339845	Up Regulation
gene3910	umuC	0.349858107	0.007159459	Down Regulation
gene3944	DR76_3645	0.00258784	1.87804E-18	Down Regulation
gene3945	DR76_3646	0	3.39818E-12	Down Regulation
gene3947	DR76_3647	0.000994811	1.43121E-53	Down Regulation
gene3948	DR76_3648	0.001860027	6.16879E-54	Down Regulation
gene3949	DR76_3649	0	2.90278E-35	Down Regulation
gene3950	DR76_3650	0	4.40985E-44	Down Regulation
gene3951	DR76_3651	0.001890251	2.96723E-32	Down Regulation
gene3952	DR76_3652	0.005551654	2.0364E-11	Down Regulation
gene3953	DR76_3653	0.001092924	3.05036E-65	Down Regulation
gene3954	DR76_3654	0.000447464	1.87583E-89	Down Regulation
gene3955	DR76_3655	0.002781491	5.45248E-29	Down Regulation
gene3956	DR76_3656	0	1.50749E-47	Down Regulation
gene3957	DR76_3657	0.004871698	1.41738E-27	Down Regulation
gene3958	DR76_3658	0	5.43298E-10	Down Regulation
gene3959	DR76_3659	0.001111319	2.30876E-74	Down Regulation
gene3960	DR76_3660	0.005274444	1.90361E-24	Down Regulation
gene3961	DR76_3661	0	1.00998E-14	Down Regulation
gene3962	v	0.002444649	2.01509E-47	Down Regulation
gene3963	u	0	8.31221E-16	Down Regulation
gene3964	DR76_3664	0.002031646	8.57988E-51	Down Regulation

gene3965	fii	0.002839036	2.4695E-30	Down Regulation
gene3967	DR76_3667	0.393440026	0.000150852	Down Regulation
gene3968	shp	0.257621091	4.10222E-07	Down Regulation
gene3972	DR76_3672	0.001190544	4.08514E-72	Down Regulation
gene3973	nul	0.001133088	2.17213E-48	Down Regulation
gene3977	DR76_3675	0.188861736	0.00012422	Down Regulation
gene3978	bor	0	8.15094E-34	Down Regulation
gene3981	DR76_3678	0	2.09295E-32	Down Regulation
gene3982	DR76_3679	0.012061485	9.28705E-17	Down Regulation
gene3984	ompD	0.001341435	5.61117E-62	Down Regulation
gene3985	DR76_3681	0	6.09157E-17	Down Regulation
gene3986	DR76_3682	0	0.00033336	Down Regulation
gene3987	rusA	0	8.85573E-19	Down Regulation
gene3989	DR76_3684	0	2.8358E-29	Down Regulation
gene3991	DR76_3685	0.002522263	9.62403E-23	Down Regulation
gene3992	ninB	0	1.52673E-29	Down Regulation
gene3993	DR76_3687	0	2.94146E-36	Down Regulation
gene3994	DR76_3688	0.001481676	1.34005E-18	Down Regulation
gene3995	DR76_3689	0.003592746	1.51145E-18	Down Regulation
gene3996	cii	0	8.43133E-30	Down Regulation
gene3997	DR76_3691	0	1.20713E-46	Down Regulation
gene3998	ci	0.001649422	2.25268E-71	Down Regulation
gene3999	DR76_3693	0	6.74859E-25	Down Regulation
gene4000	DR76_3694	0.001063358	1.04791E-66	Down Regulation
gene4002	DR76_3695	0.000926164	5.37354E-17	Down Regulation
gene4003	DR76_3696	0.004655704	3.31469E-19	Down Regulation
gene4005	DR76_3699	0.000925256	2.87672E-53	Down Regulation
gene4006	DR76_3700	0	7.37848E-23	Down Regulation
gene4007	ciii	0	0.000319654	Down Regulation
gene4009	gam	0.002635263	5.18399E-30	Down Regulation
gene4010	bet	0.00312365	1.66806E-31	Down Regulation
gene4011	exo	0.003781197	2.48115E-33	Down Regulation
gene4016	DR76_3710	0.014210948	1.07589E-08	Down Regulation
gene4017	DR76_3711	0.002571772	1.03903E-51	Down Regulation
gene4018	DR76_3712	0	2.26996E-11	Down Regulation
gene4019	DR76_3713	0	1.10774E-25	Down Regulation
gene4020	DR76_3714	0.005171468	4.55944E-19	Down Regulation
gene4021	DR76_3715	0	2.66231E-39	Down Regulation
gene4022	DR76_3716	0	3.49507E-30	Down Regulation
gene4023	DR76_3717	0	5.37532E-08	Down Regulation
gene4024	DR76_3718	0	5.8977E-19	Down Regulation
gene4025	DR76_3719	0.001976286	1.69319E-47	Down Regulation
gene4061	DR76_3752	0.149105063	5.70831E-15	Down Regulation
gene4062	DR76_3753	0.418478188	6.99168E-05	Down Regulation
gene4138	ptsG	8.728464902	2.56353E-24	Up Regulation
gene4155	rne	2.135839031	0.000109054	Up Regulation
gene4175	grxB	2.057899204	0.000383901	Up Regulation
gene4178	dinI	0.497373999	0.004178527	Down Regulation
gene4343	pgaA	0.475178387	0.000300283	Down Regulation
gene4355	putA	4.812506283	0.000732376	Up Regulation

gene4366	wrbA	2.601878538	2.35744E-06	Up Regulation
gene4501	artJ	3.519909464	0.014195931	Up Regulation
gene4539	pflD	3.626729631	0.031243134	Up Regulation
gene4635	cydB	2.139536036	0.003556127	Up Regulation
gene4663	kdpA	0.42441005	0.001987945	Down Regulation
gene4695	asnB	2.269646001	0.008922576	Up Regulation
gene4808	folD	2.077616432	0.000777812	Up Regulation
gene4864	htpG	2.113712559	0.000141863	Up Regulation
gene4900	lon	2.009305103	0.000377403	Up Regulation
gene4911	cyoC	2.059770545	0.00032376	Up Regulation
gene5017	betI	2.150907493	0.006863591	Up Regulation
gene5018	betB	3.087520379	0.006403688	Up Regulation
gene5019	betA	3.911295368	0.006862259	Up Regulation
gene512	sthA, udhA	2.164998758	0.002074238	Up Regulation
gene5196	hisD	2.122290461	0.000151859	Up Regulation
gene5197	hisC	2.539678772	3.82224E-06	Up Regulation
gene5198	hisB	2.440650057	9.94704E-06	Up Regulation
gene5199	hisH	2.725447076	1.78234E-06	Up Regulation
gene5200	hisA	2.505774066	1.16583E-05	Up Regulation
gene5202	hisIE	2.001633443	0.000490043	Up Regulation
gene5270	fbaB	2.059944484	0.000413178	Up Regulation
gene5342	parA	2.507523454	2.74327E-06	Up Regulation
gene549	hslV	2.585393975	4.65706E-05	Up Regulation
gene550	hslU	3.021277608	3.10736E-08	Up Regulation
gene557	fpr	2.312364987	3.08066E-05	Up Regulation
gene587	fdnG	2.274070487	2.92295E-05	Up Regulation
gene588	fdxH	2.144014905	0.000126113	Up Regulation
gene589	DR76_555	2.239530942	7.68047E-05	Up Regulation
gene62	fghA	2.145000688	0.000118991	Up Regulation
gene756	rbsC	2.982012468	0.013728127	Up Regulation
gene757	ccmA	2.987828472	0.007486316	Up Regulation
gene762	asnA	2.388871191	0.019935196	Up Regulation
gene778	pstS	2.125223605	0.002974614	Up Regulation
gene84	DR76_76	2.596421677	2.06966E-06	Up Regulation
gene840	uhpT	0.484434447	0.007309444	Down Regulation
gene977	hokA	0.475130914	0.004978376	Down Regulation

Table S5. Differentially expressed genes between groups B and D

Gene ID	Gene name	Foldchange(DvB)	Pval	Regulation
gene1014	fimA	2.918425277	0.025101628	Up Regulation
gene1062	manX	4.04441837	2.77142E-05	Up Regulation
gene1149	manZ	8.501310115	5.30348E-05	Up Regulation
gene1159	ptsG	4.143137567	0.000151598	Up Regulation
gene1165	manY	2.260348696	0.012635561	Up Regulation
gene1285	malT	2.030364316	0.020255312	Up Regulation
gene1287	lamB	2.281748519	0.032967829	Up Regulation
gene1506	uspF	2.196578071	0.00675467	Up Regulation
gene1531	malG	3.127081751	6.43564E-05	Up Regulation

gene1588	asnA	3.224542358	0.016718398	Up Regulation
gene1701	bssR	2.050765178	0.016136962	Up Regulation
gene1821	wrbA	2.52901171	0.007801675	Up Regulation
gene1822	malF	2.308563534	0.03007916	Up Regulation
gene1842	soxS	3.388945775	2.04861E-05	Up Regulation
gene1843	malM	2.699348325	0.001878449	Up Regulation
gene1844	malE	2.155612868	0.011683097	Up Regulation
gene198	cydB	2.216351082	0.00766115	Up Regulation
gene209	asnB	3.074219718	0.049441783	Up Regulation
gene212	feoB	2.266494667	0.021429023	Up Regulation
gene2171	uspB	2.422678639	0.001605911	Up Regulation
gene2173	malK	2.053949426	0.00728735	Up Regulation
gene2214	grcA	7.819939887	0.000722389	Up Regulation
gene2215	cydA	4.873690677	0.006194811	Up Regulation
gene2216	dmsB	4.52217502	0.000288929	Up Regulation
gene2218	dmsA	3.949498282	2.82106E-05	Up Regulation
gene2219	gcvT	8.493937668	0.000138362	Up Regulation
gene2220	adhE	4.607762611	0.006415553	Up Regulation
gene2262	aspC	4.647383824	0.001221639	Up Regulation
gene2358	crr	2.634026083	0.035548719	Up Regulation
gene2359	exbB	2.831781558	0.025187969	Up Regulation
gene2375	ibpB	2.949718944	0.007746398	Up Regulation
gene2376	mlc	2.499319635	0.016678463	Up Regulation
gene2396	glgS	2.610015264	0.031029196	Up Regulation
gene2594	fimI	23.60719297	6.53297E-07	Up Regulation
gene2595	nuoM	3.082455871	7.65023E-05	Up Regulation
gene2727	frdB	2.037712977	0.009125024	Up Regulation
gene2818	dctA	2.202110218	0.02269513	Up Regulation
gene2855	nmpC	2.209988815	0.00672135	Up Regulation
gene2902	pflD	2.020653726	0.021425197	Up Regulation
gene3127	nifJ	2.897816055	0.046615966	Up Regulation
gene3205	aspA	2.805944088	0.000249909	Up Regulation
gene3250	grxB	12.5808921	2.26458E-05	Up Regulation
gene3251	znuC	10.24931775	9.4662E-05	Up Regulation
gene3252	bssS	15.63617042	2.70507E-06	Up Regulation
gene3351	gcvH	2.644900116	0.000834235	Up Regulation
gene3374	ptsI	2.206953424	0.038376078	Up Regulation
gene339	pfkB	2.321725493	0.027125338	Up Regulation
gene3392	dcuA	2.065092433	0.017794784	Up Regulation
gene340	purA	2.677923235	0.022100395	Up Regulation
gene341	gldA	3.244937643	0.003716137	Up Regulation
gene3473	fumC	2.550300159	0.016250668	Up Regulation
gene3493	pgk	3.147212541	0.008822135	Up Regulation
gene3718	frdA	2.863677038	0.047077347	Up Regulation

gene3721	zraP	8.101818806	6.78433E-11	Up Regulation
gene3838	ndh	3.324283568	0.010103604	Up Regulation
gene4130	ptsH	2.354927391	0.010802905	Up Regulation
gene4138	fbaA	12.52365578	0.000320792	Up Regulation
gene4175	fdoI	2.826443238	0.026463539	Up Regulation
gene4179	smg	2.717495348	0.012148112	Up Regulation
gene4366	nuoJ	5.154175379	4.91812E-09	Up Regulation
gene4432	dmsC	3.268267254	0.003845864	Up Regulation
gene4461	ompR	2.261790406	0.027785945	Up Regulation
gene4462	fdoG	3.586014503	2.00281E-05	Up Regulation
gene4463	menB	3.438627697	1.13198E-05	Up Regulation
gene4526	fhuA	5.361545917	5.25961E-08	Up Regulation
gene4539	ppsA	2.894170455	0.006875399	Up Regulation
gene4635	pdhD	4.370346697	0.000153539	Up Regulation
gene4636	mug	3.869049949	9.99522E-05	Up Regulation
gene4695	gcvP	4.297574351	7.83871E-07	Up Regulation
gene490	fdoH	3.911479195	0.015830424	Up Regulation
gene531	menI	2.564998039	0.037042098	Up Regulation
gene587	zraS	2.228383996	0.019395042	Up Regulation
gene588	iucA	2.107056632	0.038767258	Up Regulation
gene589	caiB	2.288168091	0.02614012	Up Regulation
gene762	def	6.195468349	3.38743E-05	Up Regulation
gene821	proS	3.166330195	5.37732E-05	Up Regulation
gene1311	iaaA	0.370553173	0.002346096	Down Regulation
gene2000	prfH	0.22205358	1.02127E-07	Down Regulation
gene2001	xseA	0.33049386	1.0627E-05	Down Regulation
gene2082	gmr	0.382398502	0.010975532	Down Regulation
gene2083	motA	0.334520147	0.019786557	Down Regulation
gene2319	tsr	0.414359878	0.01649518	Down Regulation
gene2637	rtcB	0.477084742	0.006915879	Down Regulation
gene2655	yfiP|	0.424647556	0.010126366	Down Regulation
gene2656	rnpA	0.425149331	0.002669034	Down Regulation
gene3141	cdh	0.424046376	0.00407274	Down Regulation
gene3172	guaB	0.477224891	0.003144799	Down Regulation
gene3173	uhpA	0.413294691	0.006945745	Down Regulation
gene3246	cysP	0.360888078	0.001902678	Down Regulation
gene344	rimI	0.412742527	0.000999771	Down Regulation
gene345	holD	0.397675183	0.003044701	Down Regulation
gene346	fliZ	0.386934257	0.001177996	Down Regulation
gene347	phnC	0.409075352	0.000309781	Down Regulation
gene348	motB	0.437824958	0.000672427	Down Regulation
gene3486	cysM	0.35797259	0.003061289	Down Regulation
gene3606	cysU	0.398019163	0.004486981	Down Regulation
gene3632	gsiA	0.115069735	0.000691037	Down Regulation

gene3766	cysP	0.310526066	0.001720846	Down Regulation
gene3767	fimAI	0.29062893	2.07592E-06	Down Regulation
gene3768	cysA	0.323047476	0.000403679	Down Regulation
gene3769	rseC	0.230200538	6.86618E-05	Down Regulation
gene3787	umuC	0.479277369	0.008221257	Down Regulation
gene3910	cysW	0.387532769	0.014976606	Down Regulation
gene3984	proW	0.0011718	1.53002E-38	Down Regulation
gene3987	dusB	0.006578485	2.77129E-17	Down Regulation
gene417	maa	0.44849632	0.045635128	Down Regulation
gene418	rlmA1	0.489561902	0.004991026	Down Regulation
gene4533	mdtI	0.407777971	0.000411842	Down Regulation
gene4534	proV	0.497171568	0.004853918	Down Regulation
gene481	cys	0.391073992	0.008978829	Down Regulation
gene4880	pspB	0.362848489	4.64501E-05	Down Regulation
gene495	pspD	0.466760344	0.008591379	Down Regulation
gene5074	pspC	0.491524263	0.008297094	Down Regulation
gene5075	pspA	0.47483157	0.008096959	Down Regulation
gene564	cysD	0.455458305	0.006256359	Down Regulation
gene565	higA	0.400512222	0.001332772	Down Regulation
gene802	rusA	0.465847256	0.017801037	Down Regulation
gene837	ompD	0.441315777	0.006076432	Down Regulation
